# The education-chasing labor rush in China identified by a heterogeneous migration-network game

**DOI:** 10.1038/s41598-020-68913-3

**Published:** 2020-07-31

**Authors:** Xiaoqi Zhang, Yanqiao Zheng, Zhijun Zhao, Xinyue Ye, Peng Zhang, Yougui Wang, Zhan Chen

**Affiliations:** 1 National School of Development, Southeast University, Nanjing, 210000 China; 2 School of Finance, Zhejiang University of Finance and Economics, Hangzhou, 310018 China; 3 Institute of Economics, Chinese Academy of Social Science, Beijing, 100836 China; 4 Department of Landscape Architecture and Urban Planning, Texas A&M University, College Station, TX, 77840 USA; 5 School of Systems Science, Beijing Normal University, Beijing, 100875 China; 6 School of Statistics, Capital University of Economics and Business, Beijing, 100070 China

**Keywords:** Social evolution, Complex networks, Statistical methods

## Abstract

Despite persistent efforts in understanding the motives and patterns of human migration behaviors, little is known about the microscopic mechanism that drives migration and its association with migrant types. To fill the gap, we develop a population game model in which migrants are allowed to be heterogeneous and decide interactively on their destination, the resulting migration network emerges naturally as an Nash equilibrium and depends continuously on migrant features. We apply the model to Chinese labor migration data at the current and expected stages, aiming to quantify migration behavior and decision mode for different migrant groups and at different stages. We find the type-specific migration network differs significantly for migrants with different age, income and education level, and also differs from the aggregated network at both stages. However, a deep analysis on model performance suggests a different picture, stability exists for the decision mechanism behind the “as-if” unstable migration behavior, which also explains the relative invariance of low migration efficiency in different settings. Finally, by a classification of cities from the estimated game, we find the richness of education resources is the most critical determinant of city attractiveness for migrants, which gives hint to city managers in migration policy design.

## Introduction

Uncovering the mechanisms that govern the inter-regional migration behavior of human beings is critical for understanding and managing a wide range of issues that interest both social scientists and policy makers, from analyzing the motives and welfare change of migrants to the design of migration policies^1–8^. The increased availability of large migration data sets that capture details of migration activities creates an unprecedented opportunity to explore the motivations, consequences and patterns of migration. The early works in this field aim at identifying the overall network patterns provided the occurrence of migration^1,9–11^, modelling the human interaction dynamics that drive migration to happen^1,12–22^, and quantifying the casual relationship between various social-economic factors and migration decision^1–6,23–27^. Apart from those topics, there is a recent wave of studies focusing on unifying the migration decision-making, human interactions and the migration pattern recognition together, so as to better understand the complexity of migration. It has been shown that the classical gravity law of migration across multiple destinations^12–21^ can be derived from a destination choice model (DCM) for a variety of migrants coming from different starting locations. A more recent work^28^ shows that interactions among migrants can be added into the DCM framework, by which the DCM becomes the destination choice game (DCG) that include DCM as a special case . The so-called “route congestion” effect^11,28–30^ can be formally studied within the DCG framework, which merges the heterogeneity of migrants on their starting locations and their interactions together, helping DCG generate better fitting to the observed migration trend.

In addition to the heterogeneity for the starting locations and the interactions among migrants, the other fundamental driving force of migration is the benefits that migration can bring to migrants. The benefits of migration could depend on a variety of migrant-level social-economic factors in a complex way^1–6,23–27^. Economists and sociologists have made persistent effort in both qualitatively and quantitatively understanding the relationship between migration decision and the social-economic factors, such as the education, age, income, family wealth, gender difference and social capital^1,3,4,27,31–34^. It is shown that migration is profitable for young and poor migrants as it grants them and their later generations with the chance to chase well-paid job opportunity, better education and/or healthcare services^8,31,35–37^. On the ohter hand, migration is costly and hard to afford for the migrants with limited family wealth, low education level and lacking of social connection in the potential destinations^3,4,33,38–40^. Therefore, migration is essentially the consequence of a trade off between the benefit and cost, the social-economic factors are important as they shape the way to calculate the benefit and cost for different types of migrant. On that basis, a natural question is arisen, which factor is most influential for migration decision, is migration for chasing better education, healthcare services, job opportunity and/or the others? The other related question is whether the relationship between the social-economic factors and migration decisions can change over time or is stationary for a long period. This question is critical for multi-stage migrations, but due to the limitation in both the data and the empirical methodology, it is rarely studied in the literature. It turns out that including the migrant-level social-economic features into the migration decision process is indispensable for answering above questions, but by now only the location-level heterogeneity are formally studied in the framework of DCM/DCG, the variation of the migrant-level features has not yet been included. So, we ask: how to incorporate the migrant-level social-economic features into the DCM/DCG framework to study the migration behavior? Can the inclusion of migrant-level features help generate better forecast for the real destination selection? Will they give hint to the deep motives of inter-regional migration behavior and its dynamics?

It is not trivial to extend the location-level heterogeneity to the migrant-level heterogeneity in the DCG framework. So, in order to address above questions, we propose a population game to model the interactive migration decision in which the migrant-level features are added through a continuum feature space. With the help of adding migrant-level features, many meaningful mechanisms, other than the classical congestion effect, route congestion effect and the migration cost effect^28,39^, can be well represented in our model, such as the “subjective congestion effect”, capturing the interpersonal difference in evaluating the congestion and its dependence on migrant types. To our best knowledge, these mechanisms have not yet been formally studied in the existing literature within the context of migration, despite their usefulness in classifying and identifying the attractiveness of destinations, measuring migration efficiency and so forth. Our new model is fittable by real data through a mild modification of the game-econometric technique^41–45^. The numeric analysis of the model is based on a resume data set extracted from one most famous online job platform in China. The data supports the analysis of two-stage migrations, by which some dynamic facets of the migration pattern can be studied. We identify and compare the two-stage labor migration networks, the forecast accuracy and migration (in)efficiency are also analyzed as by-products of the model, which grant us the chance to identify the stationary nature of the migration decision pattern over time. Finally, we highlight that although the analysis in this paper is mainly based on Chinese labor migration data, our method is general and not restricted to labor force migration in China, it can be applied to analyzing human migration behavior in a wide range of settings, such as the patient transfer behavior among hospitals, international immigration behavior and the vehicle route selection issues.

The remaining sections of the paper is organized as the following. In the result section, an brief overview and visualization to the migration dataset studied in the paper are provided, followed by a formal description on the migration game model and the equilibria migration network, inefficiency index network derived from the model. On the basis of model fitting, we discuss the stationary nature of the hidden migration decision pattern behind the two-stage migration, and sketch how the migrant-level heterogeneity affects the migration patterns and the migration efficiency. In the end, we introduce a classification criterion for cities according to the inefficiency level of migration flows and briefly discuss the implication of applying the classification to our data. In discussion section, we discuss the further implication of our game model and data analysis. "Method" section introduces the technical details of the set-up of migration game model and its training procedure.

## Results

### Data description and the network structure of aggregated migration flows

We study the labor force migration of Chinese online job-seekers by a large resume dataset. The resume data is collected from Zhaopin.com that is one leading online platform for job seeking in China and has a resume database consisting of tens of million resumes filled by real job-seekers when they registered on this platform. Since the filled resumes have high probability to be viewed by HRs from the intended recruiters, the information filled by job-seekers is believed to reflect their truth and is updated in time. The questions mandatory to be answered include the gender, age, marriage status, education experience, past working experience (most job seekers on zhaopin.com has at least one job before), the previous working industries and so on, from which 77 migrant-level feature variables can be extracted (a complete statistic description of these variables in our sample can be found in the supplementary to the paper). The resume also contains the information of migrant’s hukou place, the current working place, the expected working places. Since the hukou place of job seekers can be identified as the place where they come from, the resume data set provides a two-stage OD trajectory data set for the labor force migration in China that are the stage (1): hukou place $\rightarrow$ current working place and stage (2): current $\rightarrow$ expected working place.

The data that we have the access is a purely random subsample of the full resume database which consists of resumes from 80,000 job-seekers (after dropping the records with missing values, 75,616 records are remained), the most recent update time of the resumes in this sample is by Jun. 2017. The included hukou places, working places and expected working places cover 400+ cities in China which include all the 286 prefecture-level cities and a set of lower-level cities of China, therefore we believe the subsample presents a good representative for the city-level labor force migration trend at least for the sub-population who seek job online. To avoid over-fitting, we group all the destination cities into 22 city clusters among which the first 21 city clusters are officially declared in the Chinese Statistical Yearbook (2015), the last one consists the cities that are not contained in any of the officially declared city clusters.

**Figure 1:**
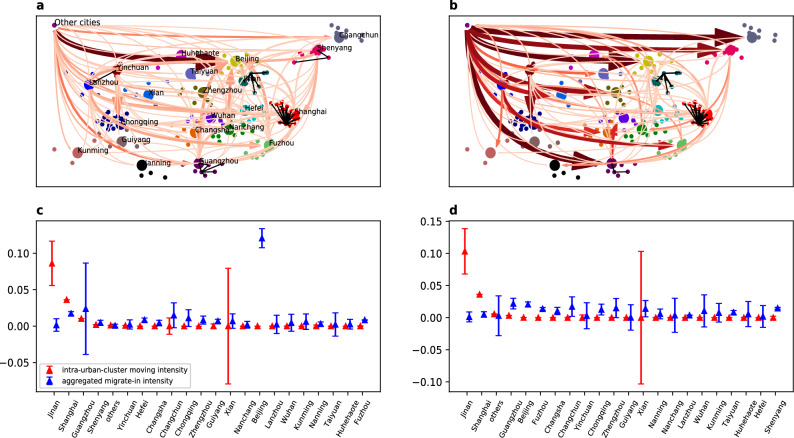
Overall migration network for the two-stage migrations (**a**,**b**) sketch the aggregated migration network formed by the mean conditional migration probability from the origin city (group) to the destination city (group). To simplify network structure, only the 178 cities included in the officially declared 21 major city clusters are plotted with their exact geographic locations (longitude v.s. latitude). All the cities out of the 21 city clusters are clapsed into one point labeled with “other cities”, the location of this point does not have any geographic sense and only the arrows linking this point with the others are meaningful. The central cities in the 21 city clusters are highlighted with their name labeled. The bold arrows between city pairs always point toward the destination city, the size, darkness and opacity of the arrow represent the value of the aggregated mean migration probability. For the simplicity of representation, the aggregated mean migration probability is calculated only for central cities of the 21 city clusters and the “other cities” through summing up the mean migration probability between all city pairs in the origin and destination city clusters, therefore all bold arrows are always link two central cities (or the “other cities”). The migration flows within each of the 21 city clusters are kept and represented as the slim arrows, their size, darkness and opacity represent the absolute value of the migration probability from the origin to the destination. (**c**,**d**) present the to-degree of every city cluster from the other city clusters and the total link weight within every city cluster (the sum of migration probabilities between all city pairs within a city cluster) for the migration network of stage (1) and (2), respectively. The to-degree reflect the attractiveness of a city cluster and the total link weight charaterize the tightness of inner-connection within each of the city clusters.

From the data, the aggregated migration network for the two stages can be calculated simply by counting the number of sampled migrants who migrate between every city pair. We can discuss the structural features of the migration network and its structural changes for the two migration stages. The two-stage networks are presented in Fig. 1a,b, where to generate clean plots, we only display the migration arrows across the 22 city clusters and the migration arrows within every city cluster. Comparing the two plots, the decentralization or multi-centralization trend is observed, and this trend can also be verified through comparing Fig. 1c,d where the blue line represents the total to-degree of every city cluster. From Fig. 1a,c, during the first stage, Beijing is the unique global flow-in center of all migrants, attracting migrants from both the other major cities (city clusters) in China, such as Shanghai, Guangzhou, and the lower-level periphery cities (grouped within “Other cities”). Although there do exist a couple of local flow-in centers such as Changchun in the north China and Shanghai in the East China, while their attractiveness is not comparable to Beijing at all. In contrast, during stage (2), multiple global flow-in centers emerge and they are detectible from comparing Fig. 1b,d. The local centers, such as Guangzhou, Zhengzhou and Xi’an, are upgrade to global centers and their attractiveness becomes in line with Beijing, which impedes the leading position of Beijing. The attractiveness of Beijing is even reduced in the absolute sense that the strong migration flows from the local centers, such as Shanghai, Fuzhou, Guangzhou and Shenyang, in stage (2), are significantly weakened or even disappears during stage (2). Finally, during stage (2), many tier-2 cities, such as Zhengzhou, Changsha and Xi’an, starts to be attractive for migrants who come from periphery cities. If we focus on the within-cluster migration trend, the difference between the stage (1) and (2) migration is also remarkable and coincides with the trend of decentralization. In fact, by the comparison between Fig. 1c,d, the internal migration intensity is strengthened significantly for two city clusters in the stage (2) that are the set of other cities and city cluster centered at Beijing. The former implies an increasing trend of the out-of-center migration^46^, while the later implies the decline of Beijing in terms of absorbing local migrants which reinforces the finding that the attractiveness of Beijing is reduced in the absolute sense.

The observed decentralization trend is highly consistent with the divergence in city development planning and migrant-related policies since 2016 between the capital city Beijing and the other major cities in the middle and western area of China. Since 2016, Beijing started to control its population scale, issue unfriendly residential policies that expel the so-called “low-end” migrants who are the low-educated, low-skilled, low-income and elderly contractor workers without registration in local hukou system. In the mean time, many tier-2 cities in the middle and western area of China, such as Xi’an, Chongqing, Chengdu, Wuhan, and cities in the Yangtze river delta area, such as Hangzhou and Nanjing, and cities in the Pearl river delta area, such as Shenzhen and Guangzhou, became migrant-friendly and relaxed the hukou restriction to attract young migrants with relatively high education level. Since the second-stage migration in our dataset only refers to the expected migration which has not yet happened in reality, the expectation of migrant workers reacts much more quickly to the policy change than their real migration decision, the structural change of the migration network between the two stages is by and large attributable to the non-homogeneous change of migration policy in different cities.

Although the overall migration network is informative, it is not complete. In reality, the migration network often varies along with migrant types, such as the education, income and age^3,34,38,47^, while the aggregated network fails to detect the difference. In addition, there are complicated interactions among migrants which also contribute to the formation of migration network^28^ but are not reflected in the aggregated network. To this end, we shall develop an empirically-fittible population game model for further investigation.

### A family of large migration game

For simplicity we focus on the origin-destination (OD) type of migration trajectories, but the framework can be easily extended to more general situations. To formulate the interactions among migrants, a large population game is established where the player set is viewed as a large random sample drawn from a continuum feature space and all the strategies that players can select are identified as the set of destination locations. The continuum feature space assumption is designed to capture the heterogeneity of players and the fact that there are always enormous players involved in the migration analysis. For short, we shall call this game model as migration game throughout the paper.

Formally, consider a family of normal-form games defined through the following four components and represented as the tuple $G(X_N\subset {\mathfrak {P}}:={\mathbf {C}}\times {\mathbb {R}}^p,{\mathbf {C}},\mu ,U)$:

A1.: Pure strategies: denote ${\mathbf {C}}=\{c_1,\dots ,c_n\}$ as the pure strategy set which consists of all possible destination locations for players.
A2.: Players: denote ${\mathbb {R}}^p$ as the *p*-dimensional feature space, every $x\in {\mathbb {R}}^p$ represents a personal feature profile that associates with a given player (type). Augmenting ${\mathbb {R}}^p$ with the origin place ${\mathbf {C}}$ forms the full set of potential players characterized by both their personal and location features, denoted as ${\mathfrak {P}}={\mathbf {C}}\times {\mathbb {R}}^p$. The set of *N* players $X_N$ is randomly drawn from ${\mathfrak {P}}$, according to a known distribution $\mu$, which can be interpreted as the population distribution.
A3.: Mixed strategy: players are allowed to take mixed strategy, the mixed strategy set is represented as the set of vector-valued function ${{\mathscr {P}}}=\{P:X_N\rightarrow S_{\mathbf {C}}\}$ where $S_{\mathbf {C}}=\{(p_0,\dots ,p_{|{\mathbf {C}}|-1})\in {\mathbb {R}}^c:\,p_i\ge 0,\sum _{i=0}^{|{\mathbf {C}}|-1}p_i=1\}$ is the $|{\mathbf {C}}|-1$ dimensional simplex, $|{\mathbf {C}}|$ is the cardinality of ${\mathbf {C}}$. Then, for every destination $j\in {\mathbf {C}}$, the *j*th coordinate projection $P_j(i,x)$ will be the probability that a player $(i,x)\in X_N$ selects to migrate to *j* under the mixed strategy *P*. Without loss of generality, we assume $P\in {{\mathscr {P}}}$ is smooth up to a certain order with respect to $x\in {\mathfrak {P}}$, i.e. *P* is the restriction of a smooth function onto $X_N$, which implies that two players who are similar to each other in both of origin and features should make similar choice of strategies to some extent.
A4.: Utility: denote $u^p$ as the pure strategy utility function of players, it takes the following form for a given player $x_+=(i,x)\in X_N$ and a pure strategy profile ${\mathbf {s}}=\{s_{x'_+}\in {\mathbf {C}}:x_+'\in X_N\}$:
  1 $$\begin{aligned} u^p(x_+,j,{\mathbf {s}}_{-x_+})= F\left( \frac{1}{N-1}\sum _{x_+'\not = x_+}I(s_{x'_+}=j)T(x_+,x_+')-g\left( x_+,j\right) \right) \end{aligned}$$
  where ${\mathbf {s}}_{-x_+}$ is a pure strategy combination executed by players other than the decision player $x_+$, *I* is the indicator function. *F* is a continuous function; *T* is a pairing weight function valued in the unit interval [0, 1] which, for a given player $x_+$, describes which group of competitors, namely $\{x_+'\in X_N:T(x_+,x_+')>0\}$, will be taken into account and how influential, measured by the value of function $T(x_+,\cdot )$, the competitors are for $x_+$; *g* is interpreted as the ideal population ratio that the destination location should have, which is a kind of private information for every player and the features of every player can affect this quantity in a certain way. The utility for mixed strategy is calculated from Eq. (1) by the standard von-Neumann–Morgenstern expected utility theory, denoted as $U_{vNM}$.

For every pure strategy *j*, Eq. (1) postulates that the utility it brings for every decision player depends on the difference between the actual population ratio [represented as the partial sum term in Eq. (1)] and the ideal population ratio [represented as the value of function *g* in Eq. (1)]. This difference can be interpreted as a measure for the degree of congestion in the destination city. As both the ways to count the actual population ratio and the ideal population ratio in Eq. (1) are allowed to vary from player to player, the congestion effect studied in this paper is essentially the subjective congestion effect, which gives full respect to the migrant-level heterogeneity, and is more flexible, including the widely studied congestion effect and the route congestion effect^11,28–30^ as special cases. For the actual population ratio of a given decision player, only those players within the target group are counted, while which player is in the target group is completely determined by the pairing function $T(x_{+},\cdot )$ evaluated at the feature type of the decision player. Hence, the pairing function *T* encodes the relationship among players, it can be interpreted as the continuous-version adjacency matrix of a player-to-player network. This player-to-player network is closely related to the concept of “social capital” in the studies of social network, including it into the utility function helps establish a quantitative connection between the adjacency matrix of the social network among migrants and that of the migration network among regions^22^. This connection is important to understand the formation of migration trajectories. To our best knowledge, our work is the first attempt to connect the two networks together at the adjacency-matrix level.

The ideal population ratio is also conditional on the feature of every player. This is because different players often disagree with each other, the disagreement can come from both the difference in personal characteristics (represented through their feature vector *x*) and the different original cities^1,28^. For instance, local residents with higher education level are more likely to survive in expensive meta-cities than those migrants with lower education level, consequently, the former group of people tend to evaluate a greater *g* for big cities, while the later group would assign a greater *g* to small and cheap cities when all others equal.

Throughout the application of the current paper, we simply assume $F(x)=-x^2$, and *g* is parametrized through the logistic form

2 $$\begin{aligned} g(i,x,j|\theta _g)=\frac{1}{1+\exp (-\theta _{g,1}^\top \cdot x_i-\theta _{g,2}^\top \cdot x-\theta _{g,3}^\top \cdot x_j)}, \end{aligned}$$

where *x* is the person-level feature vector for a given player, $x_i$ and $x_j$ are the city-level feature vectors associated with the origin city *i* and destination city *j*, respectively. $\theta _g \, (:=(\theta _{g,1},\theta _{g,2},\theta _{g,3}))$ consists of the three coefficient vectors associated with the feature $x_i$, *x* and $x_j$. Under this specification, we assume that every job-seeker obtain utility from migration to city *j* through comparing the difference between his/her subjective optimal population ratio of city *j* measured by *g*(*i*, *x*, *j*) and the actual ratio of job seekers that select to migrate into *j*. Using quadratic functional form for *F*(*x*) captures that the utility is maximized if and only if the actual population ratio is just the ideal ratio, both overwhelming and unsaturatedness would lower down the attractiveness of a destination (notice that the quadratic form of *F* can be generalized to any functional form preserving the preference order that would have no impact on the current analytic and numerical results, for instance the quadratic form can be replaced by another function that has unique peak value and is symmetric with respect to the peak). This specification captures the dynamics of migration in many real world settings (e.g. the labor force migration game, the vehicle route selection game), similar utility functions are also used in the literature^39^.

For the pairing function and *T*, we consider two alternatives that are the constant function $T\equiv 1$ and the binary($\{0,1\}$)-valued function with $T\left( (i,x),(i',x')\right) =1$ if and only if $i=i'$. The later option describes the peer effect behind migration by which migrants only take their “peers”, the migrants who come from the same origin, into account. A preliminary analysis shows that the constant function dominates the “peer-effect” pairing function in forecast accuracy for migration decision at both migration stages, therefore, we would only focus on the simpler setting $T\equiv 1$ in the following sections. One reason for the relative disadvantage of the peer-effect *T* might be that the destination locations in our data are the city-level locations which are too-large to allow the peer effect to work.

Finally, note that under the adjacency matrix interpretation of *T*, the constant pairing $T\equiv 1$ is equivalent to that the social network that drive the formation of migration network is essentially a fully-connected network. In the other words, every job-seeker in the game will put equal weight to the decision of all the other players. This assumption is a bit weird as in reality job seekers is not possible to know all their competitors. But on the other hand, this assumption is equivalent to ask the job-seekers read the officially published population data of every city, which is not that unrealistic any more. It is definitely possible to set a more subtle form of the pairing function *T* so as to better reflect the impact of social networks among job seekers, but that is beyond the scope of both the data and the current study, we leave it for future studies.

#### Equilibrium, migration network and efficiency

The actual migration should happen in the way that every migrant in the system can only move to the destination that maximizes their utility given the choice of the others, which can be perfectly captured by the Nash equilibrium of the migration game. In fact, we can assume the following without loss of generality.

##### Assumption 1

Given *n* randomly sampled migrants $\{x_{+,i}:\,i=1,\dots ,n\}$, there exists an Nash equilibrium mixed strategy $\{P^*_E(x_{+,i},\cdot ):\,i=1,\dots ,n\}$ such that the observed *n* OD trajectories $\{OD_i:\,i=1,\dots ,n\}$ are independent random samples with each $OD_i$ drawn from the law of $P^*_E(x_{+,i},\cdot )$.

This assumption sets the actual migration trajectory as a random consequence with the randomness governed by a set of probability laws that are derived as a latent mixed-strategy Nash equilibrium of a proper migration game. The randomness set-up is to capture the impact of individual heterogeneities that are unobservable and beyond the scope of the observed feature *x*. We show in the method section that under Assumption 1 the proposed migration game can be fitted by real OD-trajectory data through a constrained maximum likelihood procedure, and a fast algorithm is provided to generate consistent inference for both the equilibria migration probability and the underlying game that migrants actually play.

For an Nash equilibrium, $P_E$, of the migration game $G(X_N,{\mathbf {C}},\mu ,U)$ that generates the observed trajectory data, we can always construct the equilibria migration network which is representable as the following directed weighted adjacency matrix:

3 $$\begin{aligned} M_{x}=\{P_{E}(i,x,j)\}_{i,j\in {\mathbf {C}}},\,\,x\in {\mathfrak {P}}. \end{aligned}$$

The *ij*th entry of $M_{x}$ is the probability that a player would like to migrate from his/her origin to the given destination, which can be interpreted as the migration intensity for a certain type of migrants *x*. The dependence of $M_{x}$ on player’s feature *x* reflects the heterogeneity of migration networks across different types of player, which is of the great interest in this study.

Apart from the adjacency matrix, a set of quantitative indices can be constructed from combining both of the game structural information and the equilibria migration probabilities. For instance, the following index is constructed as an analogue to the measure of energy conservation in physics, which is the product of two deviation quantities and presents a quantitative measure for the inefficiency degree of the migration dynamics governed by the equilibria migration networks (3):

4 $$\begin{aligned} DE_x=\left\{ DE_{x,ij}^1\cdot DE_{x,ij}^2\right\} _{i,j\in {\mathbf {C}}},\,\,x\in {\mathfrak {P}} \end{aligned}$$

where

5 $$\begin{aligned} DE_{x,ij}^1= & {} \left( P_{E}(i,x,j)-\int _{{\mathbf {C}}\times {\mathfrak {P}}/\{(i,x)\}} P_{E}(i',x',j)T\left( (i,x),(i',x')\right) d\mu (i',x')\right) \end{aligned}$$

6 $$\begin{aligned} DE_{x,ij}^2= & {} \left( \int _{{\mathbf {C}}\times {\mathfrak {P}}/\{(i,x)\}} P_{E}(i',x',j)T\left( (i,x),(i',x')\right) d\mu (i',x')-g(i,x,j)\right) \end{aligned}$$

In fact, under the set-up $F(x)=-x^2$, a great positive $DE_{x,ij}$ implies two situations: (1) the location *j* has been overwhelming (positive $DE_{x,ij}^1$) in the view of the player with type *x* coming from *i*, but the player is still highly likely to move in (positive $DE_{x,ij}^2$); and (2) the location *j* has the potential to grow up (negative $DE_{x,ij}^1$) in the view of the player while he/she is less likely to move in (negative $DE_{x,ij}^2$). Therefore, the positive $DE_{x,ij}$s would polarize the population distribution across cities, which would induce the coexistence of resource overuse and over in-flow, and cause the migration inefficiency. In contrast, negative $DE_{x,ij}$ implies an equalization potential for the population dynamics which we think as the representative for migration efficiency.

### Heterogeneity of migration networks cross migrant types

**Figure 2:**
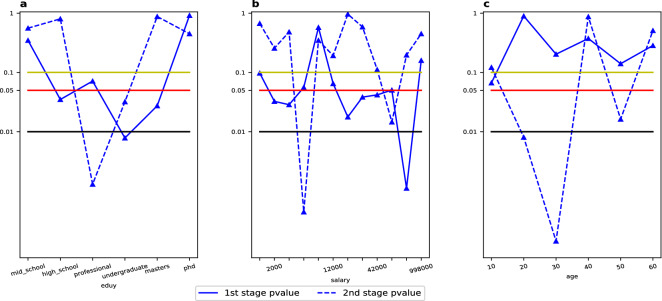
Heterogeneity degree of migration networks. The figures sketch the variation trend of P values at both migration stages for the null-hypothesis that the overall and type-specific migration networks are identical to each other for a series of migrant types. (**a**) Presents the P values variation along with the increasing of migrant’s education level, (**b**) with the increasing of monthly salary, and (**c**) with the increasing of migrant’s age.

The proposed game model is applied to infer the type-specific migration networks for a variety of migrant types. In practice, it is needed a set of migrant-level features and city-level features to fit the migration game model and derive the equilibria migration networks. In our case, there are 77 migrant-level features and 21 dummy variables accounting for the heterogeneity induced by the 22 city clusters. To avoid the multicolinarity for migrant-level features, we adopt the principal component analysis (PCA) method to generate a few principal features that covers 99% of total variance of the migrant-level variables. The number of the remained PCA features is 15. We will run the estimation based on the 36 (= 15 + 21) feature variables for the two-stage migration data. The result is presented in Fig. 2. by which we discuss the structural change of the migration network against the two migration stages and three classes of migrant-level features, including the education level, age and income. The three classes of features are selected according to the existing literature in which these features are most critical to migration decision^3,34,38,47^.

In Fig. 2, we first test the significance of the heterogeneity of migration networks against a variety of migrant types. Within each migrant type, we calculate the type-specific migration network through marginal integration of the estimated equilibria networks over the domain of migrant features provided that the given dimension of *x* (education, income and age respectively) is fixed at the desired type value. To show the significance of the heterogeneity of type-specific migration network we conduct the Z test against the null hypothesis that the given type-specific migration network is indifferent from the overall network. Under the null hypothesis, the entry-wise difference between the estimation of the type-specific and the overall network should be a random variable drawn from a zero-mean distribution. In Fig. 2, we report the Z test result for all different education, income and age types and for both stages of migration. From Fig. 2, a majority portion of migrant types are heterogeneous at the 0.1 confidence level and around a half of types have distinct migration network at the 0.05 confidence level. This result verifies the necessity to include migrant-level features into the analysis of migration networks. Among the three classes of migrant types, it is found that for education types, the migrants with undergraduate degree at stage (1) and migrants with professional school degree at stage (2) tend to deviate most significantly from the aggregated population; for income types, the extreme high-income group (with monthly salary above 84,000 Chines yuan) at stage (1) and the middle-class (with monthly salary around 6,000 Chinese yuan) at stage (2) deviate most; for age types, the youngest migrants (older than 10 but younger than 20) at stage (1) while the middle-aged adults (between 30- and 40-year-old) at stage (2) deviate most. Based on these results, we can roughly summarize such a rule that at the initial stage of migration, the migrants with some extraordinary features (such as high education, high income and extreme low age) are more likely to behave differently, while in the expected stage of migration, the migrants that take the most proportion within the whole population (such as middle education, income and age class) tend to deviate significantly. This contraction is quite interesting and deserves further explanation in the future studies.

In sum, Fig. 2 validates such a viewpoint that the migration network is not universally homogeneous for different migrant types. The heterogeneity is rooted deeply in the social-economic background for particular types of migrant, therefore, to understand the real mechanisms that govern the labor force migration behavior, including the migrant-level heterogeneity is indispensable. This fact proves, once again, the usefulness of the model proposed in this paper. Finally, note that Fig. 2 only characterizes the significance of the deviation between type-specific and overall migration networks, it does not reveal how the deviation happens. In the supplementary, we present more details of the deviation through plotting the difference migration network, which is formed by entry-wisely subtracting the type-specific migration probability from the overall migration probability (see Supplementary Figs. S1, S2 and the attached discussion).

### Forecast accuracy and the stationary nature of migration dynamics

In this section, we validate the inference made by our population game model in terms of its forecast accuracy. The accuracy is evaluated at both migration stages by which a comparison of the decision pattern between the two stages is conducted. We divide the full sample into a training sample which consists of 68,000 randomly picked resumes from the full sample and a testing sample that include the remained 7,616 resumes. Due to the large size of the training sample, we apply a bootstrap method to fulfil the estimation. We randomly partition the training sample into 10 subsamples with equal sample size and run the fast estimation algorithm (see the “Method” section) on each of these subsamples. Based on the estimation result of each subsample, we generate a set of forecast on the test sample and calculate the accuracy, the final accuracy is aggregated through averaging over all the subsample accuracies. A comparison is made on the forecast accuracy for our game-based method (GBMLE), the kernel-density forecast (see “Method” section) and the forecast by the aggregated adjacency matrix (Fig. 1a,b) that is calculated through counting the proportion of migrants starting from every given city to all the other destination cities^9,10^.

**Figure 3:**
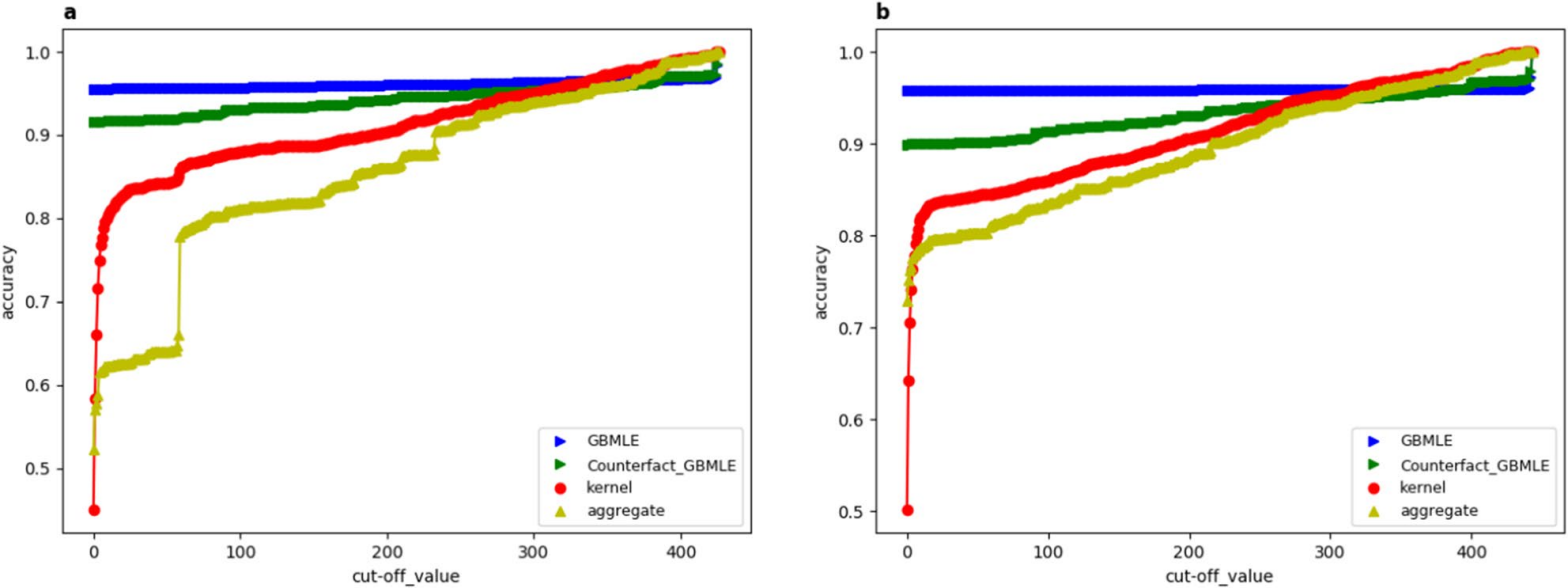
Forecast accuracy for two-stage migrations In the two figures, the horizontal axis represents the cut-off value *k* for the top *k* forecast introduced in the main text, the vertical axis represents the accuracy rate, therefore, each curve in the two figures presents the variation trend of out-sample forecast accuracy of a given method along with the parameter *k*. (**a**,**b**) Represent the stage (1) and (2) migration, respectively. The red and yellow lines represent the forecast accuracy generated by the kernel method and aggregated method. The blue line is the accuracy curve generated by the proposed GBMLE method. The blue line in both (**a**,**b**) are generated by the same set of training and testing sample, which means the trainning sample used to estimate game parameters are drawn from the same population to the testing sample used to evaluate the accuracy. In the other words, in (**a**) both the training and testing sample to generate the blue line are drawn from the stage (1) migration data, and in (**b**) both the trainning and testing sample are drawn from the stage ii) migration data. In contrast, the green lines in (**a**,**b**) sketch the accuracy of GBMLE by using counter-fact trainning and testing sample. In (**a**) the game parameters are estimated from the trainning sample drawn from the stage (2) migration while the accuracy is evaluated by the stage (1) testing sample, in (**b**), the opposite holds, the game parameters are estimated from the stage (1) training sample while the accuracy is evaluated by the stage (2) testing sample.

Since there are about 400 destination locations and every migrant only selects one as its destination, generating forecast in this case is a typical multi-class classification problem. Following the literature^48,49^, we adopt the set-valued forecast method to generate destination forecast from the computed migration probability and evaluating its accuracy. Given the migration probability for every destination location, we first sort all destinations in the descending way by the probability values. For a given positive integer *k*, we consider the top *k* forecast as the set of first *k* destinations in the sorted sequence. The top *k* forecast is thought to be accurate if and only if the true destination falls into the top *k* set. In Fig. 3, we plot variation trends of the forecast accuracy along with the parameter *k* for the migration probabilities calculated by three different methods.

From Fig. 3a,b, the proposed GBMLE method generates the best forecast accuracy for almost all *k* in both the two stages of migration, while the kernel forecast outperforms the aggregated forecast in general. This observation verifies our theoretical prediction that the GBMLE method outperforms the kernel method by incorporating the game structural information, while they both dominates the aggregate method because they both distinguish the migrant-level heterogeneity while the aggregated method does not.

In addition to the overall accuracy, it is also impressive that the GBMLE forecast accuracy can reach its maximum even when *k* is fairly small, while the average accuracy exceeds 95% at $k=1$. In the other words, there is at most 5% chance by which the maximum-a-posteri forecaster would mistakenly pick one destination out of 400 alternative destinations. In contrast, the maximum-a-posteri forecast by the kernel method and aggregate method would have 50% chance of making mistakes. This difference is striking and it demonstrates the power of adding the game structural information in increasing the forecastibility.

By the green lines in Fig. 3a,b, we make a counter-fact experiment. The purpose is to test the forecastability of the 1st-(2nd-)stage migration trajectories by the estimated 2nd-(1st-)stage migration game model. In details, when generating the green line in Fig. 3a, the model parameters are the same as those used in generating the blue line in Fig. 3b which are estimated from the migration data at stage (2), rather than from the “correct” stage (1). The relation between the green line in Fig. 3b and the blue line in Fig.  3a is the same. Compare the green with the blue line in Fig. 3a(b), we find that although the forecast accuracy by GBMLE is a bit lower after replacing the model parameters, it is still significantly higher than the alternative methods (they are not affected by the parameter change). Moreover, the accuracy for the maximum-a-posterior forecast by GBMLE in the parameter-change case can still be around 91%, which has been good enough and is not much different from the case that “correct” parameters are used. Therefore, it can be expected that the underlying mechanisms driving the observed migration are not significantly distinct for the two stages, and migrants’ behavior is highly consistent over time. This finding is very striking, because according to the model set-up, this finding implies a kind of “where-they-stand-depends-on-where-they-sit” decision pattern: given that the migrant-level features are identical for two migrants A and B while their original cities, CA and CB, are different, if A migrated to CB during the first stage, then the decision pattern of A in choosing the second-stage destination would be analogous to the decision pattern of B in choosing the first-stage destination while differs from A itself during the first-stage decision. In the other words, the labor migration dynamics revealed by our data follows a Markovian decision process, i.e. when all others equal, only the current destination matters the migration in the future, the home town does not. This finding presents a positive evidence for the use of markovian models in describing human migration dynamics^33,50^. Finally, note that the observed “where-they-stand-depends-on-where-they-sit” decision pattern and the markovian property relies heavily on controlling the migrant-level heterogeneity. Without including the social-economic features of migrants, the feature-dependent migration probabilities reduce to the entries in the aggregated migration adjacency matrix which are significantly distinct for the two migration stages by Fig. 1, therefore, the markovian properties is not detectable unless the migrant-level heterogeneity is involved. This fact validates the usefulness of our model.

### Migration inefficiencies and polarization of migrants distribution

**Figure 4:**
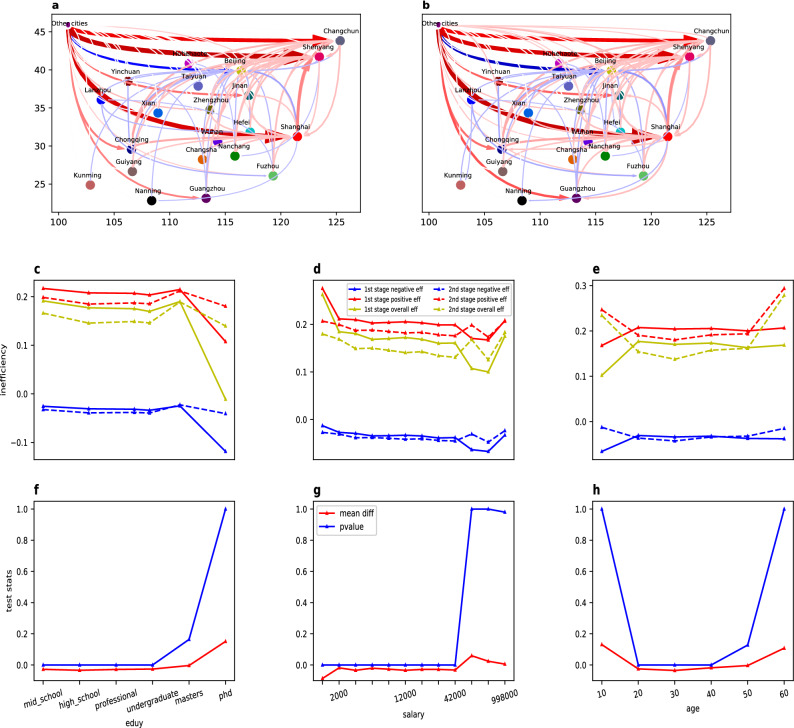
Migration efficiency across different migrant groups (**a**,**b**) plot mean of the migration inefficiency network (4) for the stage (1) and (2) migration, respectively, where the arrow always points toward the destination of the migration flow, the size, darkness and opacity of the arrow represents the absolute mean value of the inefficiency measure, and the blue-colored arrow implies negative mean inefficiency measure and the red-colored implies positive mean inefficiency measure. The inefficiency is aggregated up to the city-cluster level and every city cluster is represented by their central city. (**c**–**e**) Present the average of the mean inefficiency measure over all city pairs within a variety of education, income and age groups, respectively. Within each of the subfigures, we also decompose the ineffiency measure into the positive part and negative part, and take average for each of these two parts. (**f**–**h**) Sketch the gap of the average inefficiency measure over all city pairs between the migration stage (1) and (2), and the left-tail pvalues of the null hypothesis that the average inefficency measure is indfference between stage (1) and (2), where the smaller pvalue means the greater confidence to take the alternative hypothesis that the average inefficiency is lower for stage (2) than stage (1).

We ask whether the observed migration is efficient and how the degree of efficiency (measured by Eq. 4) varies in reaction to the migrant types and migration stages. To this end, we plot the overall inefficiency network [calculated by entry-wisely integrating equation (4) over all migrant-level features i.e. $DE_{{{\mathscr {X}}}}:=\{\frac{1}{|{{\mathscr {X}}}|}\sum _{x\in {{\mathscr {X}}}}DE_{x,ij}\}$ with $|\cdot |$ representing the cardinality, ${{\mathscr {X}}}$ the set of all migrants having the given feature] in Fig. 4a,b for the two migration stages from which it is visualized that the spatial distribution of the positive/negative signs of the inefficiency arrows and the arrow strengths (corresponding to the sign and absolute value of entries of the inefficiency matrix (4)) are almost identical for both of the two migration stages. The stability of the migration inefficiency is also verified by Fig. 4c–e where we plot the aggregated two-stage migration inefficiency against different education, income and age types. It is shown that for the major portion of migrants in our sample, which are the migrants with education level no more than undergraduate, monthly income no more than 42,000 and age older than 20 and younger than 50, the variation trend of the overall inefficiency against the increasing of education, age and income are parallel for the two stages, while the 2nd-stage overall efficiency are systematically higher than the 1st-stage by a constant which reflects as the aggregated inefficiency measure (Eq. 4) in Fig. 4c–e is lower in stage (2). In order to measure the significance of the difference, we conduct the left-tail Z test and the result in Fig. 4f–h shows that at the 5% confidential level, the inefficiency measure in stage (2) is significantly lower than that in stage (1) for the majority portion of migrants. In addition, we apply ANOVA to test the significance of the parallel difference trend, it shows that within the same range of education levels as above, we cannot reject that the 2nd-stage inefficiency curve in Fig. 4c differs from the 1st-stage inefficiency curve by a constant even at the 20% confidential level, the same holds for age and income as well which provide positive evidence for the parallel trend.

The increasing migration efficiency is quite intuitive and consistent with the job searching theory^51^ since the first-stage migration helps accumulate more information of the potential destinations that facilitates migrants to make rational decision. The relative invariance of migration efficiency against the variation of multiple migrant-level features is quite surprising. One possible explanation for this observation is the invariance of the migration game which is supported by Fig. 3, because the identical game set-up implies the same functional form of *g* which defines the ideal population ratio of each destination and also consists of a key component of the inefficiency measure (4). Hence, the invariance of *g* provides a stablizer for the two-stage migration inefficiency.

Finally, it is observed from Fig. 4c,d a decreasing trend of migration inefficiency along with the increasing of education and income at least for the majority population (education level less than the master’s degree and monthly salary less than 42,000). This observation implies that high-educated and well-paid migrants tend to make more rational decision in selecting destination which agrees with the literature^52^ that high education can increase the decision rationality and the fact that the high income are often the consequence of high education.

### Classification of destinations and education-chasing behind migration dynamics

From Fig. 4, it is clear that in both of the two migration stages, the overall efficiency is poor (reflected as the inefficiency measure is significantly positive for both stages). Then it is natural to ask what causes the inefficient migration. To answer that question, we notice that there are two sources leading to the positive inefficiency measure by definition equation (4): (1) a migrant is more likely to migrate to a destination than the average even if the given destination is already over-sized in his/her view; (2) a migrant is less likely to migrate to a destination than the average while the destination can better off if more migrants can come in. Similarly, the negativity of the inefficiency measure also arises from two sources that are exactly opposite to the two above. Given that, all destination cities can be partitioned into four classes: (I) the **np** cities that consist of the unsaturated cities (with $\sum _{i}DE_{x,ij}^2<0$) with positive aggregated flow-ins $(\sum _{i}DE_{x,ij}^1>0$); (II) the **nn** cities that consist of the unsaturated cities (with $\sum _{i}DE_{x,ij}^2<0$) with negative aggregated flow-ins $(\sum _{i}DE_{x,ij}^1<0$); (III) the **pn** cities that consist of the oversized cities (with $\sum _{i}DE_{x,ij}^2>0$) with negative aggregated flow-ins $(\sum _{i}DE_{x,ij}^1<0$); and (IV) the **pp** cities that consist of the oversized cities (with $\sum _{i}DE_{x,ij}^2>0$) with positive aggregated flow-ins $(\sum _{i}DE_{x,ij}^1>0$).

**Figure 5:**
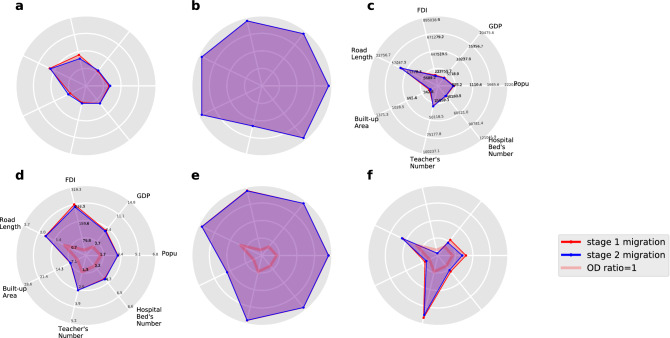
Features of four city classes (**a**–**c**) present the radial plot for the means of the seven city-level features for the **nn**, **pn** and **np** class of cities respectively for both stages of migration; (**d**–**f**) present the radial plot for the mean OD ratios of the seven city-level features for the **nn**, **pn** and **np** class of city pairs respectively for the two stages of migration. Because the **pp** class contains no city nor city pair for both stage (1) and (2) migration, the relevant radial plots are missing. During the classification, to avoid the data noise, we trimed those very small valued $DE^1_{x,ij}$ and $DE^2_{x,ij}$ in the sense of setting $DE^1_{x,ij}$($DE^2_{x,ij}$) as 0 when their absolute value is less than 0.001, then the resulting city pair *ij* is discarded as noisy point and will not be rendered into any of the four classes.

We ask what is the major feature of the four classes of city, how do they differ from each other and how is the difference related to the inefficient migration pattern? To this end, we consider the seven city-level features: population, GDP, foreign direct investment (FDI), total road length, city building area, the total number of teachers in all primary and middle schools and the total number of beds in all hospitals. The seven features are devoted to capture the social-economic development level of a city, among which the number of teachers and beds are proxies to the education and healthcare resources, which are two most important public goods and very influential to migration decision^35–37,53^. The city-level feature data is collected from Chinese Statistical Yearbook (2015) which are only available for the 178 cities in the 21 city clusters, so the aggregation is also taken on that basis. Fig.  5a–c present the mean of the seven features within each city class where the city classification and mean features are calculated for the overall inefficiency measure (integrating $DE^1_{x,ij}$, $DE^1_{x,ij}$ with respect to all *i* and *x*) and for both migration stages.

Since the inefficiency measure (Eq. 4) is defined on the level of migration flow between city pair, the four classes of cities can even be extended to the set of all migration flows, which becomes four classes of ordered city pairs. For each of the city-pair classes, we compute the OD ratio of the seven features which are ratios formed by dividing the value of the origin city at a certain feature dimension by that of the destination city. The OD ratios convey more information than the absolute value of city-level features because they encode the comparison between the origin and the destination cities. The mean OD ratio for the four city-pair classes and its variation along with education, age and income are plotted in Fig.  5d–f (in Supplementary Fig. S10, we plot the radial graph for the mean features and OD ratios for the per capita value of the six features other than population, which is the quotient by letting the feature value divide the population, which shows a qualitatively identical pattern to the Fig. 5).

The geometric shape of the feature area within each of the radial plots in Fig.  5 are quite identical for both migration stage (1) and (2), this observation supports, once again, the invariance of the migration game. For the mean features and OD ratios of the city classes, some remarkable properties can be concluded. First, compared to the unsaturated cities (the **nn** and **np** class in Fig. 5a,c), the oversized cities (the **pn** class in Fig. 5b) are significantly greater in both migration stages and in all of the seven city-level features. This finding is not trivial because except for the 22 city-cluster dummy variables, no city-level social-economic feature is included when fitting the migration game. In the other words, the classification by the migration data can have a perfect match to the real social-economic gap between the “large” and “small” cities even though the social-economic data of cities are missing from the classification process. This result establishes a natural link between the migration behavior and the city “size” which suggests a novel way to describe the “largeness” or “smallness” of a city.

Second, the **np** class differs from the **nn** class mainly in the stock of education resources measured by the number of teachers in primary and middle school and the **np** class of cities tend to own better education resources. This fact is robust for both migration stages, and can even be reinforced through comparing Fig.  5d,f where the relative advantage on the teacher’s number is much more significant for those **np** city pairs. The right-tail *Z* test is conducted to measure the significance of the finding for a variety of migrant types, the result shows that the teacher’s number is always significantly higher in the **np** class at the 0.05 confidential level for almost all situations (see Suplementary Fig. S3). In contrast, the other features, such as the healthcare resources measured by the bed number, do not differ much between the **nn** and **np** classes (see Supplementary Figs. S4–S9). The extraordinary role played by education resources in attracting migrants results deeply from the fact that the education resources are immobile which makes migration the only way to chase better education^54^. Although the healthcare resources have the similarly immobile nature, it cannot impact the migration as much as the education resources because living temporally is fully feasible to take use of the healthcare services in a different city, there is no need to induce observable migration flow. In contrast, schools will not provide service unless migration really happens. In that sense, the education resources are more immobile than the healthcare resources, therefore offering better education services could form a long-lasting comparative advantage of a city in attracting migrants, this advantage will not leak out and be “freely” used by non-migrants.

Finally, the comparative disadvantage of **nn** class in education resources also provides an explanation to the overall inefficient migration pattern. In fact, around 4/5 of all ordered city pairs are contained in the **nn** class, which dominates all the other three classes and constitutes the main portion of the inefficient migration arrows in Fig. 4a,b As the **nn** class of city (pairs) are caused by the relative shortage of education resources in the destination cities, it is reasonable to claim that the imbalanced spatial distribution of education resources is a major driving force of the overall inefficient migration. This finding is consistent with the existing studies on the relationship between Chinese labor migration and the education system^40^, and provides positive evidence for the argument that the distortion on education induces the distortion of population migration.

## Discussion

To summarize, we study a large migration game model that can be fitted with real data through a constrained maximum likelihood procedure. From the game model, the conditional migration probability among the set of destinations can be naturally identified with a mixed-strategy Nash equilibrium of the game. The migrant-level heterogeneity is easily embedded into the game via the feature space of migrants, as a consequence, the resulting equilibria migration behavior is represented as a family of type-specific migration networks. Through applying the model to a two-stage Chinese labor-force migration dataset, we find that although the network structures are significantly distinct for two migration stages, the underlying migration game is essentially unchanged. Given the stationary game set-up, even if a migrant can select different destinations at different stages, the difference can only be induced by the different starting cities, which implies a “where-they-stand-depends-on-where-they-sit” decision pattern. In the macro view, this decision pattern suggests the shuffling of migrants’ distribution be the main source of the network structural change for the 2nd-stage migration in relative to the 1st-stage one. The staionarity of the game set-up also helps stablize the migration inefficiency measure whose distributions along with the spatial coordinate and the major migrant features turn out invariant qualitatively for the two migration stages. In addition, we test the heterogeneity of migration network against a variety of education, age and income types. The result shows that the migrant types can significantly alter the structure of migration networks. This result is quite robust, indicating the necessity of incorporating migrant-level feature into migration network analysis. The decentralization trend is found widely existing in the 2nd-stage migration, whose existence, although, is robust for various migrant types, they occur in quite different ways that are migrant-type dependent. Finally, based on the migration model, a four-fold classification of cities can be carried out by the averaged inefficiency. A statistic analysis on the class-level features suggests that the overall inefficient migration trend observed from the data is rooted deeply in the unbalanced spatial distribution of the primary education resources which drives migrants away from those unsaturated cities even if moving into these cities can induce the potential utility increasing.

Among the existing literature, Ref.^28^ made an important step toward understanding the migration network formation that underlie modelling the human interaction, migration decision and the destination-level heterogeneity together. The main finding in Ref.^28^ is that the inclusion of interaction among migrants coming from different source locations can significantly increase the forecast accuracy for the overall migration trend, and the gravity law of human migration can be naturally derived from such an interaction framework. However, as the analysis in Ref.^28^ is focused only on the inclusion of the heterogeneity of the starting locations, it cannot distinguish the heterogeneity induced by migrant types. Therefore, still little is known about the microscopic mechanisms that drive the formation of the migration network, which makes the model in Ref.^28^ only capable of generating volume forecast between city pairs rather than the forecast on the migration-decision level. In contrast, the main contributions of our paper are: (1) to propose a general methodology based on population game model and game econometric method to analyze the migrant-level decision making, migration network formation and the impact of migrant-level feature types, (2) to reveal empirically the change of migration network structures along with the migration stages and migrant types, the unchanged nature of the underlying set-up of the migration game and the migration efficiency pattern, (3) to identify a variety of city types that play significant roles in shaping the migration network and determining the migration efficiency.

One of the main findings in this paper is that chasing for better chance of education is a major motive for migrants to give up the cities that are currently unsaturated and can grow better if more migrants move in, the dominance of such a choice mode within the population also contributes to the overall low migration efficiency. Therefore, our result suggests that city managers should focus on accumulating the high-quality education resources if they are going to attract more migrants into their place. For instance, more fiscal expenditure can be allocated to building primary and middle school facilities, and provide well-paid teaching positions for qualified candidates. Another way is to relax the hukou registration policy for elite teachers and attract the high-quality teachers from the outside world. Our work provides a general framework for incorporating migrant-level heterogeneity and interactions into migration decision making and migration network formation, several promising extension can be built on this work. First, the migration game studied in this paper is essentially a complete-information game, which requires that every player has perfect knowledge on the other’s actual move. This assumption is a bit too strong in real world. In the future studies, the classical game-theoretical concepts of partial information or mis-belief should be included^55,56^. In addition, the current paper only studies the origin-destination trajectory. In reality, there exist many more complicated types of trajectory, to model them, properly designed dynamic games should be studied. Finally, we highlight that whenever the data is available, the non-trivial social network structure can be added into the analysis and the connection between migrant-level social network and the city-level migration network can be established, which would convey more insightful information for understanding the real motives of human migration behavior.

Finally, the recent pandemic of COVID-19 across the world has intrigued a large body of literature^57–60^ to re-think the connection among regional development, cross-regional migration trend, the risk of infectious disease outbreak and its control. Notably, the analysis and methodological framework of the current paper is closely related with the discussion on this topic. Comparing to the existing studies, our migration game model and the training technique pay more attention to the population-level interaction, its impact on migration trend and the connection with the real-world data, which might bring extra insights into the field of epidemic modelling where the migration is often supposed to be rule-based and the analysis is pure-theoretical or based on synthetic data, which cannot capture the impact of strategic behavior on the risk of virus outbreak and the complexity from the real world. But on the other hand, there is no direct link between infectious disease outbreak and migration decision in the current model, how to extend the migration game model to include the impact of infectious disease into the utility function becomes an open question and is left for future studies.

## Methods

### Kernel-density estimator for migration network

There exists a natural non-parametric estimator to the underlying Nash equilibrium mixed strategy, which can be derived by the kernel density method as below:

7 $$\begin{aligned} {\hat{P}}_E(i,x,j)=\frac{\sum _{l=1}^n K_h^{d_x}(x-X_l)I(o_l=i,t_l=j)}{\sum _{i=1}^n K_h^{d_x}(x-X_l)I(o_l=i)} \end{aligned}$$

where *n* is the number of observed OD trajectories, $X_l$ is the feature vector for the *l*th migrant, $o_l$ and $t_l$ are the origin and target location of the migrant, $K_h^{d_x}$ is the $d_x$ dimensional Gaussian kernel function with bandwidth *h*, $d_x$ is the dimension of the feature vector *x*, *I* is the indicator function.

Under Assumption 1, the classical non-parametric statistic theory^61^ guarantees that the estimator ${\hat{P}}_E$ converges to the true Nash equilibrium mixed strategy in Assumption 1 (denoted as $P^*_E$) in the rate of $O(\frac{1}{\sqrt{nh^{d_x}}})$. The consistency of estimator ${\hat{P}}_E$ makes it useful for the design of data-adaptive fast algorithm in the following sections.

Although the estimate in Eq. (7) is consistent for an infinite sample of the migration game, in the finite sample case, it will lead to under-estimate of $P^*_E(i,x,j)$ for those target location *j* for which $P^*_E(i,x,j)>0$ but *j* is selected very rarely by migrants who originate in location *i*. Meanwhile, ${\hat{P}}_E(i,x,j)$ over-estimates $P^*_E(i,x,j)$ for the *j*s that are out of the best response set. Obviously, the bias of ${\hat{P}}_E(i,x,j)$ for finite samples comes mainly because it only utilizes the observed data, the game structure is not included at all.

### Estimation procedure

Constrained maximum likelihood algorithm can apply to estimate both of the equilibrium mixed strategy and the parameters of the game. Notice that under Assumption 1, the likelihood function of the observation migration OD trajectory data can be derived from the Nash equilibrium mixed strategy, by which a constrained maximum likelihood estimation procedure can be derived as below and the procedure facilitate the simultaneous estimation of both the equilibria migration probability for each migrants (i.e. the Nash equilibrium strategy $P_E$) and the unknown structural parameter $\theta$(:=$\theta _g$ in Eq. (2)) of the migration game.

8 $$\begin{aligned} \begin{gathered} \max _{{\mathbf {p}}_n,\theta }L({\mathbf {p}}_n,\theta )\\ s.t. \,\,\, {\left\{ \begin{array}{ll} {\sum }_{j\in {\mathbf {C}}}U_{vNM}(x_+,j,{\mathbf {p}}_{-i})p_{i,j}=\max \left\{ U_{vNM}(x_{+,i},j,{\mathbf {p}}_{-i}):\,j=1,\dots ,M\right\} \\ {\sum }_{j=1}^{m}p_{i}(j)=1\\ p_{i}(j)\ge 0\\ i=1,\dots ,n;\,j=1,\dots ,m. \end{array}\right. } \end{gathered} \end{aligned}$$

where the log-likelihood function *L* is specified as below:

9 $$\begin{aligned} L(p_1,\dots ,p_n,\theta )=\sum _{i=1}^{n}\sum _{j\in {\mathbf {C}}} log(p_{i,j})\cdot I(t_i=j) \end{aligned}$$

$U_{vNM}$ is von-Neumann–Morgenstern expected utility of a given mixed strategy derived from Eq. (1); $p_{i,j}=P(x_{+,i},j)$ is a short-hand notation for the probability that the *i*th player $x_{+,i}$ will migrate to *j* under the given strategy *P*; $p_{i}=(p_{i,j})_{j\in {\mathbf {C}}}$ is the vector representing a given mixed strategy for player $x_{+,i}$; ${\mathbf {p}}_n=(p_1,\dots ,p_n)$ is the collection of mixed strategy for all players, ${\mathbf {p}}_{-i}=(p_j:j\not = i)$ is the profile of the strategies of all the other players than the player $x_{+,i}$; $t_i$ is the observed target location of the *i*th player. The estimator ${\hat{P}}^*_E$ and ${\hat{\theta }}$ for equilibria migration probability and game structural parameters is derived from solving the problem (8). Here we use the function notation ${\hat{P}}^*_E$ rather than the vector notation $\hat{{\mathbf {p}}}$ to express the estimator for the equilibria migration probability, because as we shall show in the last section that the estimated equilibria probability can be viewed as a function which can evaluate at an arbitrary player *x* even if *x* is not contained in the fitting sample.

It is remarkable that the constrained maximization problem (8) includes both the information from the observed migration trajectories and the structure of migration games in the sense that the result estimator must maximize the probability of observing the actual migration trajectories, meanwhile the probability has to constitute a Nash equilibrium of a proper migration game. It turns out that the combination of both sources of information leads to a more accurate estimator than the classical kernel-density estimator to the equilibria migration probability, which replies solely on the observed trajectory data and fails to utilize the game structure.

Theoretically, the optimization problem (8) is solvable, but in practice, searching for an optimal solution is infeasible for a large amount of players and pure strategies, because in that situation the calculation of the expected utility $U_{vNM}$ and the searching for feasible domain of the constraints in Eq. (8), which is equivalent to searching for all Nash equilibrium mixed strategies for a giant population game, are not computationally tractable. In addition, the objective function in Eq. (8) takes all migration probabilities of all players as parameters, which would diverge along with the number of players, the relevant computation is neither tractable for large population game. Finally, except for all the computation issues, the effectiveness of the estimator derived from Eq. (8) may also suffer from the missing data. This is because in reality the observed migrants are always a subset of all potential migrants, i.e. the actual player ser $X_n\subset X_N$ with $n<<N$. According to Eq. (1), the pure strategy adopted by the unobserved migrant can always impact the utility of the observed migrants, which makes the missing observation a series issue. The next two sections are devoted to resolve the missing-data issue and the computation issue, respectively.

### Deal with unobserved migrants

As pointed out previously, the estimation procedure (8) has to face the potential bias induced by unobserved migrants. In this section, we will gives a validation of the procedure (8) that guarantees the effectiveness of the procedure (8) even if unobserved migrants exists. As a by-product, an asymptotic formula can be derived for the von-Neumann-Morgenstern expected utility from Eq. (1) which gives a simple and fast way to compute $U_{vNM}$ when the number of players is huge. In fact, the following holds and the proof is presented in the supplementary to this article:

#### Proposition

*Suppose* *T*, *g* *are bounded functions*, *F* *is continuous. If we denote* $U_{vNM}(x_{+},P)$ *as the von-Neumann–Morgenstern expected utility of the mixed strategy profile* *P* *constructed from equation* (1), *and define* $U(x_{+},P)$ *as the following:*

10 $$\begin{aligned} U(x_+,P)=\sum _{j\in {\mathbf {C}}} F\left( \frac{1}{N-1}\sum _{x_+'\not = x_+}P(x_{+}',j)T(x_+,x_+')-g\left( x_+,j\right) \right) P(x_+,j) \end{aligned}$$

*where* $P(x_+,j)$ *is the probability that player* $x_+$ *goes to destination* *j* *under strategy* *P*, *then the following hold uniformly for all* $P\in {{\mathscr {P}}}$:

11 $$\begin{aligned} \lim _{N\rightarrow \infty }\max _{x_+\in X_N}|U(x_+,P)-U_{vNM}(x_+,P)|=0. \end{aligned}$$

The Proposition 4.3 is a direct consequence of the law of large number. It states that when the number of player *N* is large, we can think of the game as specified through the utility (10) which, compared to the classical von-Neumann–Morgenstern expected utility, is much easier to compute for large *N*. Therefore, from now on we will concentrate on the game specified through ${{\mathbf {A}}}{\mathbf {1}}$–$\mathbf {A3}$ and the mixed strategy utility (10).

Also notice that by $\mathbf {A3}$, every mixed strategy is essentially corresponding to a smooth function on ${\mathfrak {P}}$. Then, by a similar argument to Proposition 4.3, the smooth function property guarantees the uniform convergence of the utility (10) as $N\rightarrow \infty$. The convergence is crucial to the statistic inference of the game model based merely on partial observation of the player set. Because it states that even if only a subsample of players are observable, the difference of utility induced by removing those unobservable players is negligible, as long as the number of players in the observed partial game is large enough and the play of the partial game is coherent in the sense that every mixed strategy for the partial game is just a restriction of the strategy of the full game onto the observable player set. In fact, the convergence of utility also induces a convergence statement for the Nash equilibriums of the game, which is a bit too technical, so we leave the discussion to the supplementary.

With the help of the convergence result, we can make statistic inference for both the migration network and the migration games, because we can safely consider the partially observed migration game as an identically independently distributed (i.i.d.) sample of an underlying full game, and the observed equilibria trajectories of the partial game is just an i.i.d. sample that follows the law of an Nash equilibrium mixed strategy within the full game. On that basis, the standard statistic techniques are applicable to infer the true Nash equilibrium and the true game being played from the observed trajectories, which forms the theoretical foundation of the algorithm design in the following sections.

### Fast algorithm

Solving a constrained optimization problem of the form Eq. (8) needs to repeatedly search within the feasible domain postulated by the constraints. But the particular constraints (8) makes it challenging to identify the feasible domain, as the constraints come from the definition of Nash equilibriums of the migration game. By definition, Nash equilibrium condition is equivalent to a nonlinear equation system with *n* equations where *n* is the number of trajectories in the input data. When the data size is giant, solving such an equation system is infeasible. Meanwhile, in the objective function, the number of parameters increase along with the data size, which makes it unavoidable to search a high-dimension parameter space that is computationally intractable. Therefore, we have to figure out some way to reduce the dimension of the problem and fasten the speed of searching for Nash equilibrium by proper approximation tricks.

In this section, we presents a data-adaptive algorithm that utilize the observed trajectory data to simplify the procedure of solving the Nash equilibrium and convert it to an one-shot convex optimization problem. Through solving the convex optimization problem, we can represent the equilibria mixed strategy profile $\mathbf {p}_n$ as a function of the parameter vector $\theta$, and then solve the unconstrained maximum likelihood problem to derive the final estimator for both $\theta$ and ${\mathbf {p}}_n$.

Notice that when the number of players, *n*, is large,

12 $$\begin{aligned} \frac{1}{n-1}\sum _{i'\not =i}p_{i',j}T(x_{+,i},x_{+,i'})\sim \frac{1}{n-1}\sum _{i\not =i'} I(t_{i'}=j)T(x_{+,i},x_{+,i'}) \end{aligned}$$

for the true equilibria strategy ${\mathbf {p}}_n$. Then, we can use this asymptotic equivalence together with the asymptotic relation in Eq. (10) to speed up the computation. Formally, we can replace the terms of the form $\frac{1}{n-1}\sum _{i'\not =i}p_{i',j}T(x_{+,i},x_{+,i'})$ in equation (10) with $\frac{1}{n-1}\sum _{i\not =i'}I(t_{i'}=j)T(x_{+,i},x_{+,i'})$ and then replace $U_{vNM}$ in the first line constraint of Eq. (8) with the expression (10), then the feasible domain of the migration probability $p_i$s for every player $x_{+,i}$ under the Nash equilibrium constraint (8) is just the set of solution to a linear optimization problem given the parameter $\theta$, which can be easily expressed as below:

13 $$\begin{aligned} \left\{ p_{i}\in S_{{\mathbf {C}}}:p_{i,j}>0 \text { iff }j\in \arg \max \left\{ u^p(x_{+,i},j,{\mathbf {s}}^*_{-i}):\,j=1,\dots ,M\right\} \right\} \end{aligned}$$

where $u^p(x_{+,i},j,{\mathbf {s}}^{*}_{-i})$ is the pure strategy utility in Eq. (1) with ${\mathbf {s}}^*_{-i}$ being the observed pure strategy of the other players than *i*.

Then the first line constraint in Eq. (8) can be approximated by the solution to the following convex optimization problem:

14 $$\begin{aligned} \begin{gathered} \min _{p_i}\left\| {\hat{P}}_E(x_{+,i})-p_i\right\| ^2\\ s.t. {\left\{ \begin{array}{ll}p_{i,j}\ge 0\\ {\sum }_jp_{i,j}=1\\ p_{i,j}=0 \text { if }j\not \in \Sigma _i(\theta ) \end{array}\right. } \end{gathered} \end{aligned}$$

where $\Sigma _i(\theta )= \arg \max \left\{ u^p(x_{+,i},j,{\mathbf {s}}^*_{-i}):\,j=1,\dots ,M\right\}$ and ${\hat{P}}_E$ is the non-parametric estimate in Eq. (7). $\Sigma (\theta )$ depends on $\theta$ through the dependence of $u^p$ on $\theta$.

The problem (14) is a classical convex optimization problem with the strict concave objective function, this kind of problem has a unique solution and can be solved fastly. Notice that the constraints in Eq. (14) include the game structural information ($\theta$) through restricting that the migration probability is positive for a location if and only if the location is contained in the support set of the best response to ${\mathbf {s}}^*_{-i}$ .

Denote ${\mathbf {p}}^*(\theta )=(p^*_1(\theta ),\dots ,p^*_n(\theta ))$ as the optimal solution vector to the problem (14) for all players, then plugging ${\mathbf {p}}^*(\theta )$ into Eq. (9) yields a refined likelihood function that only depends on $\theta$. The final estimator ${\hat{\theta }}$ is then the solution to the unconstrained maximization problem with the objective function (9) and control variable $\theta$. Given the optimal solution ${\hat{\theta }}$, the estimator to the Nash equilibrium strategy is given as $\hat{{\mathbf {p}}}={\mathbf {p}}^*({\hat{\theta }})$. Notice that given ${\hat{\theta }}$, the estimate to the equilibrium migration probability can even be extrapolated to players who are not contained in the observed sample. In fact, given an arbitrary player $x_{+}$, the problem (14) can always be solved for $x_{+}$ and the fixed ${\hat{\theta }}$, so we can denote ${\hat{P}}^*_E(x_{+})$ as the estimator to the equilibria migration probability of player $x_{+}$ no matter whether $x_{+}$ is already in sample.

#### Remark

The data-adaptive construction of ${\mathbf {p}}^*$ is the key to reduce the dimension of the searching space for the optimization problem (8) which makes the dimension of feasible domain of the constraint (8) or (9) not depend on the number of observations any more. Meanwhile, this construction can accommodate both the game structure and the data, the resulting uniqueness of ${\mathbf {p}}^*(\theta )$ helps identify the “best” Nash equilibrium from the potentially existing multi-equilibriums. Consequently, it is no longer needed to search for the entire Nash equilibrium set as did in the reference^45^, which is almost impossible for a game with a large amount of players. Finally, the use of the data-oriented equivalence relation (12) helps reduce the repeated searching for Nash equilibrium to an one-shot convex optimization problem, which also contributes significantly to reduce the computation load.

#### Remark

It turns out the estimator ${\hat{P}}^*_E$ is more efficient than ${\hat{P}}_E$ in terms of kicking out those dominated destinations. Under mild conditions, it easily verifies that ${\hat{P}}^*_E$ has the oracle property:

*Oracle Property: There exists an* *N* *such that for every player* $x_{+}$, *if we denote* ${{\mathscr {S}}}_{x_+},\,\hat{{{\mathscr {S}}}}_{x_+}\subset {\mathbf {C}}$ *as the best response set to the equilibria mixed strategy profile* $\{P^*_E(x'_+):\,x'_+\not =x_+\}$ *and* $\{{\hat{P}}^*_E(x'_+):\,x'_+\not =x_+\}$ *by the other players, respectively, then* ${{\mathscr {S}}}_{x_+}\equiv \hat{{{\mathscr {S}}}}_{x_+}$ *for all* $n>N$.

The oracle property of the estimator ${\hat{P}}^*_E$ is remarkable that offers the finite convergence of the best response set under the Nash equilibrium $P_E^*$. The finite convergence property is not possible in general for the kernel density estimator (7). In fact, the finite convergence property of our estimator comes from its efficient utilization of the information provided by the game structure. Including the game structure through Eq. (14) guarantees an uniformly non-vanishing utility gap between those dominated pure strategies and the best response strategies under the true $P_E^*$. The non-vanishing property makes the utility gap detectable even for a large finite sample. In contrast, without referring to the game structure, the kernel density estimator have to prudently assign some positive weight to both the best-response and dominated strategies. The finite convergence property also implies a better forecast performance under finite sample, which is demonstrated in the numerical experiment in the next section.

## Electronic supplementary material

Supplementary information 1 (pdf 1527 KB)
