# Supplementary material for: The education-chasing labor rush in China identified by a heterogeneous migration-network game
Source: Sci Rep. 2020 Jul 31;10:12917. doi: 10.1038/s41598-020-68913-3 (PMC7395130; doi:10.1038/s41598-020-68913-3)
Supplement: Supplementary file 1 — Supplementary information 1 (pdf 1527 KB) [file 41598_2020_68913_MOESM1_ESM.pdf]

Supplementary to "The education-chasing labor rush in China identified by a heterogeneous migration-network game"

Xiaoqi Zhang*[1], Yanqiao Zheng†[2], Zhijun Zhao‡[3], Xinyue Ye§[4], Peng Zhang¶[3], Yougui Wang‖[5], and Zhan Chen**[6]

[1]*National School of Development, Southeast University*

[2]*School of Finance, Zhejiang University of Finance and Economics*

[3]*Institute of Economics, Chinese Academy of Social Science*

[4]*Urban Informatics-Spatial Computing Lab & College of Computing, New Jersey Institute of Technoloy*

[5]*Department of System Science, Beijing Normal University*

[6]*School of Statistics, Capital University of Economics and Business*

March 18, 2020

*Email: xiaoqizh@buffalo.edu

†Email: zhengyanqiao@hotmail.com; Corresponding Author.

‡Email: zhaozj@cass.org.cn

§Email: xinyue.ye@njit.edu

¶Email: jjs-zhangpeng@cass.org.cn

‖Email: ygwang@bnu.edu.cn

**Email: xiaoqizh@zufe.edu.cn

Technical proof for the migration game model

Proof for the proposition in method section of the main manuscript

We present the proof for the following proposition stated in the subsection "Method-Fast Algorithm" of the main manuscript.

Proposition 0.1. *Suppose T, g are bounded functions, F is continuous. If we denote $U_{vNM}(x_+, P)$ as the von-Neumann-Morgenstern expected utility of the mixed strategy profile P, and define $U(x_+, P)$ as the following:*

$$U(x_+, P) = \sum_{j \in \mathbf{C}} F\left(\frac{1}{N-1} \sum_{x'_+ \neq x_+} P(x'_+, j) T(x_+, x'_+) - g\left(x_+, j\right)\right) P(x_+, j) \tag{1}$$

where $P(x_+, j)$ is the probability that player x_+ goes to destination j under strategy P, then the following hold uniformly for all $P \in \mathcal{P}$:

$$\lim_{N \to \infty} \max_{x_+ \in X_N} |U(x_+, P) - U_{vNM}(x_+, P)| = 0. \tag{2}$$

Proof. It suffices to show the as the number of players $N \to \infty$, for each destination,

$$\sup_P \left| F\left(\tfrac{1}{N-1} \textstyle\sum_{x'_+ \neq x_+} P(x'_+, j) T(x_+, x'_+) - g\left(x_+, j\right)\right) - F\left(\tfrac{1}{N-1} \textstyle\sum_{x'_+ \neq x_+} s_{x'_+, j} T(x_+, x'_+) - g\left(x_+, j\right)\right) \right| \to_p 0 \tag{3}$$

given that the pure strategy indicator $s_{x'_+, j} = 1$ have the probability $P(x'_+, j)$. In (3), $\to_p$ stands for the convergence in probability.

Since the sequence of player set X_Ns are randomly drawn from the feature space $\mathcal{P}$ under the default probability distribution μ, (3) follows naturally from the following provided that F is continuous:

$$\sup_P E\left(\frac{1}{N-1} \sum_{x'_+ \neq x_+} s_{x'_+, j} T(x_+, x'_+) - \frac{1}{N-1} \sum_{x'_+ \neq x_+} P(x'_+, j) T(x_+, x'_+)\right)^2 \to 0 \tag{4}$$

By randomness of x_+s and $s_{x'_+, j}$ given $P(x_+, j)$, the boundedness of T and the fact that P is uniformly bounded within $[0, 1]$, the convergence of the (4) is just a consequence of the uniform law of large number.

In addition, by the same logic, it is easy to verify the following convergence result:

$$\sup_P \left| F\left(\frac{1}{N-1}\sum_{x'_+\neq x_+} P(x'_+,j)T(x_+,x'_+) - g\left(x_+,j\right)\right) - F\left(\int_{\mathcal{P}/\{x_+\}} P(x'_+,j)T(x_+,x'_+)d\mu - g\left(x_+,j\right)\right)\right| \to_p 0 \quad (5)$$

□

Continuous migration game, existence of the Nash equilibrium and its asymptotic relationship with the discrete migration game

Note that the migration game stated in the main manuscript has a continuous version, which takes the entire feature space and the set of original cities as the player set. Consequently, there are a continuum of players characterized by their features and origins in the continuous migration game, and the continuous game can be considered as the limit of an increasing sequence of the finite population game with the number of players diverging to infinity. Formally, we can record the continuous migration game as the tuple $G_c(\mathfrak{P} := \mathbf{C}\times\mathbb{R}^p, \mathbf{C}, \mu, U)$. In addition to the player set, G_c is defined in exactly the same way as the G_N in the main manuscript except for the two modification on the mixed strategies and utility function:

A3'. Mixed strategy: players are allowed to take mixed strategy, the mixed strategy set is represented as the set of vector-valued function $\mathcal{P} = \{P : \mathbf{C} \times \mathfrak{P} \to S_{\mathbf{C}}\}$ where $S_{\mathbf{C}} = \{(p_0, \ldots, p_{|\mathbf{C}|-1}) \in \mathbb{R}^c : p_i \geq 0, \sum_{i=0}^{|\mathbf{C}|-1} p_i = 1\}$ is the $|\mathbf{C}|-1$ dimensional simplex, $|\mathbf{C}|$ is the cardinality of $\mathbf{C}$. Then, for every destination $j \in \mathbf{C}$, the jth coordinate projection $P_j(i,x)$ will be the probability that a player (i,x) selects to migrate to j under the mixed strategy P. Without loss of generality, we assume $P \in \mathcal{P}$ is smooth up to a certain order with respect to $x \in \mathfrak{P}$, which implies that two players who are similar to each other in both of origin and features should make similar choice of strategies to some extent.

A4'. Utility: denote U as the utility function of players, it takes the following form for a given player $x_+ = (i,x)$ and strategy P:

$$U(x_+, P) = \sum_{j\in\mathbf{C}} F\left(\int_{\mathbf{C}\times\mathfrak{P}/\{x_+\}} P(x'_+,j)T(x_+,x'_+)d\mu(x'_+) - g\left(x_+,j\right)\right) P(x_+,j) \quad (6)$$

where F is a continuous function; T is a pairing function which, for a given player x_+, describes which group of competitors, namely $\{x'_+ \in \mathbf{C} \times \mathfrak{P} : T(x_+,x'_+) \neq 0\}$, will be taken into account when making decision; g is interpreted as the ideal population scale that the destination location should have, which is a kind of private information for every player and

the features of every player can affect this quantity in a certain manner.

As promised in the subsection "Method-Deal with unobserved migrants" of the main manuscript, we present the following proposition regarding the existence of the mixed-strategy Nash-equilibrium for the continuous version of the population game in the main manuscript, the proposition also provides an asymptotic relationship between the large population game introduced in the main manuscript and the continuous version of the game in the limit case. One important implication of the proposition is that even if there exist unobserved migrants, the statistical inference based merely on the observed sample is still asymptotically correct as long as the observed sample is sufficiently representative and sufficiently large in sample size. This implication provide a solid foundation for the empirical study of our paper.

Proposition 0.2. *(1). Given a continuous migration game $G(\mathfrak{P} := \mathbb{R}^p, \mathbf{C}, \mu, U)$, if we suppose every mixed strategy P is allowed to be a L^2 function from $\mathbf{C} \times \mathbb{R}^p$ to the $S_{\mathbf{C}} \subset \mathbb{R}^{|\mathbf{C}|}$ and define the best response set for P as*

$$\Sigma(P) := \left\{ \begin{array}{l} P' \text{ is a } L^2 \text{ mixed strategy}: \\ \sum_{j \in \mathbf{C}} u_j(P, T(x_+, \cdot), g, F) P'(x_+, j) = \max_{j \in \mathbf{C}} \{u_j(P, T(x_+, \cdot), g, F), \forall x_+ \in \mathbf{C} \times \mathbb{R}^p\} \end{array} \right\}$$

where $u_j(P, T(x_+, \cdot), g, F) = F\left(\int_{\mathbf{C} \times \mathfrak{P}/\{x_+\}} P(x'_+, j) T(x_+, x'_+) d\mu(x'_+) - g(x_+, j)\right)$. Then, the set of L^2 mixed strategy is compact convex within the topological vector space $L^2(\mathbf{C} \times \mathbb{R}^p, \mathbb{R}^{|\mathbf{C}|}, \mu)$ with respect to weak topology and the set-valued map Σ is upper hemicontinuous, therefore the Nash equilibrium exists as the fixed points of Σ.

(2). For every Nash equilibrium P_E of the continuous game, there always exist a sequence of finite discrete game $G_N(X_N \subset \mathbf{C} \times \mathbb{R}^p, C, \mu, U_N)$ $N = 1, \ldots,$ a sequence of positive numbers $\varepsilon_1, \varepsilon_2, \ldots$ that decrease to 0 and a sequence of strategies $\{P_{N,E} : N = 1, \ldots\}$ such that for each N, $P_{N,E}$ corresponds to a ε_N-Nash equilibrium of game G_N, then $P_{N,E} \to P_E$ in Banach norm.

(3). If there exists a sequence of of Nash equilibriums $P_{N,E}$s associated with an increasing sequence of finite discrete game G_Ns (i.e. the player set of G_N is always is subset of G_{N+1}) such that the left hand side of (2) converges and the sequence of $P_{N,E}$s satisfy the continuity condition that for every player $x_{+,i}$, every $\epsilon > 0$ there exists a $\delta > 0$ and a N such that $\|x_{+,k} - x_{+,i}\| < \delta \longrightarrow \|P_{N',E}(x_{+,k}) - x_{+,i}\| \, \forall N' > N$, then there exists a unique continuous Nash equilibrium P_E that is the limit of the sequence $P_{N,E}$s in Banach norm.

Proof for Proposition 0.2. In this proof, we first show that the best response function Σ

is a upper hemi-continuous set-valued function with respect to the weak topology on the $L^2(\mathcal{P}, \mathbb{R}^{|\mathbf{C}|})$.

Notice that by the definition of the weak topology and the upper hemi-continuity on the infinite dimensional topological vector space, the function Σ is upper hemi-continuous if

Condition 0.3. for a finite sequence of vector-valued functions in $L^2(\mathcal{P}, \mathbb{R}^{|\mathbf{C}|})$, denoted as $\{l_1, \dots, l_m\}$, and a sequence of positive number $(\varepsilon_1, \dots, \varepsilon_m)$ such that

$$\left| \int_{\mathcal{P}} P' \cdot l_i d\mu \right| < \varepsilon_i, \forall P' \in \Sigma(P), \forall i, \tag{7}$$

there always exists another sequence of $(l'_1, \dots, l'_{m'}\}$ and $(\varepsilon'_1, \dots, \varepsilon'_{m'})$ such that whenever a P'' satisfies (7) with l_i, ε_is replaced by l'_i, ε'_is, all $Q \in \Sigma(P'')$ satisfies (7) as well.

To verify the above continuity conditions, we first assumes that $\forall P' \in \Sigma(P)$ and all $x_+ \in \mathcal{P}$ the support set of $P'(x_+, .)$ is not the entire $\mathbf{C}$. Under this assumption and the condition that μ has compact support, it can be easily checked that there exists a small $\varepsilon > 0$ such that whenever

$$\sup_{x_+} \left| \int_{\mathcal{P}} (P - P') T(x_+, \cdot) d\mu \right| < \varepsilon,$$

the following holds

$$\min_{j \in \sigma(P)} u(x_+, P', j) > \max_{j \notin \sigma(P)} u(x_+, P', j) \tag{8}$$

which implies that $\Sigma(P') \subset \Sigma(P)$. Then, the condition (0.3) holds as long as we take $\varepsilon'_i \equiv \varepsilon/2$ and $l'_i = T(x_{+,i}, \cdot)$ for $i = 1, \dots, m'$ with $\{x_{+,i} : i = 1, \dots, m'\}$ satisfying for every $x_+ \in \mathcal{P}$ $\sup_{i=1,\dots,m'} \|T(x_+, \cdot) - T(x_{+,i}, \cdot)\|_\infty < \varepsilon/2$. Under the requirement on T in the Proposition 0.2, the sequence of $\{x_{+,i} : i = 1, \dots, m'\}$ always exists. This completes the proof for upper hemi-continuity of Σ. The convexity and weak compactness of the $\Sigma(P)$ is trivial. Consequently, by the Kakutani fixed point theorem on infinite dimensional topological vector space, fixed point exist for Σ which verifies the existence of Nash equilibrium for the continuous migration game.

The statement ii) is a direct consequence of the conclusion of Proposition 0.1 and the fact that the set of all smooth functions are dense in $L^2(\mathcal{P}, \mathbb{R}^{|\mathbf{C}|})$ with respect to the norm topology. For the statement iii), the continuity constraint guarantees the convergence of the sequence of Nash equilibriums for finite games G_N to a continuous functions which is also contained in $L^2(\mathcal{P}, \mathbb{R}^{|\mathbf{C}|})$ due to the compact support of μ, finally the limit function is a

Nash equilibrium of the limiting continuous game because of the uniform convergence result in Proposition 0.1. □

Statistic summary for the resume data sample

We present the sample distribution of 8 key quantitative variables of the resume dataset as in table 1-8. The variables include the education, age, gender/marriage status, monthly salary, the number of previous jobs, work experience (years), the number of words used to describing the past work experience and the current job status. There are many other non-quantitative feature variables in the resume data characterizing migrant types such as the text-valued variables documenting the self-evaluation, past working experience and education experience. They are not statistically summarizable, so we adopt the nature language processing techniques (such as the LDA method for topic mining) to pre-process the text-valued variables and extract 48 quantitative variables related with different latent topics, which, combining with the 9 key variables (the gender and marriage status are separated as two variables) and the 20 industry dummy variables introduced below, consist of the 77 migrant-level features used for fitting the migration game in the main manuscript. These variables, although, capture some personal features of every migrant, they are not easily interpretable in the usual sense. So we won't summarize them here.

Table 1: Distribution of education level

Education level of migrants	%
Middle school	0.81
High school	9.59
Profession school	37.47
Bachelor	50.8
Master	1.3
PhD	0.03

Table 2: Distribution of gender and marriage status

Gender/Marriage status	%
Male	138
Male(divorced)	0.6
Male(unmarried)	17.1
Male(married)	17.2
Female	14.5
Female(divorced)	0.4
Female(unmarried)	15.7
Female(married)	20.7

Next, there are 53 industries in total that Zhaopin.com provides to characterize migrants' working professions. Based on key words matching, the 53 industries can be assigned into the 20 industries officially classified by the National Bureau of Statistics of China. The table

Table 3: Distribution of past job numbers

# previous jobs	%
0	1.6
1	11.9
2	15.8
3	21.8
4	20.4
5	13.4
6	7.1
7	4
8	1.8
9	1.1
≥10	1.1

Table 4: Distribution of work experience

Work experience (years)	%
0	1
1	4.1
2	3.5
3	4.2
4	5.9
5	5.2
6	5.8
7	5.9
8	6.3
9	6.8
10	6.9
11	5.9
12	5.3
13	4.4
14	3.9
≥15	24.7

Table 5: Distribution of income

Salary (yuan/month)	%
Not disclosed	24.8
1,000-2,000	0.2
2,000-4,000	7.5
4,000-6,000	14.2
6,000-8,000	12.9
8,000-10,000	10.4
10,000-15,000	12.5
15,000-25,000	10.2
25,000-35,000	4.7
35,000-50,000	1.7
50,000-70,000	0.5
70,000-100,000	0.2
>100,000	0.1

Table 6: Distribution of age

Age	%
15-20	0.06
20-25	7.34
25-30	25.22
30-35	28.5
35-40	22.04
40-45	9.9
45-50	4.97
50-55	1.45
55-60	0.42
Older than 60	0.1

9 presents the joint distribution of migrant education and their working industry (calculated based on the 20 official industry) and makes a comparison between the joint distribution extracted from the resume data sample and the 2015 census data provided by the National

Table 7: Distribution of the number of description words

# words in the description of the past working experience	%
0-100	12.2
100-200	8.6
200-300	7.6
300-400	6.9
400-500	7.1
500-600	5.8
600-700	5.3
700-800	5
800-900	4.5
900-1000	4.1
>1000	64.6

Table 8: Distribution of job-seeking status

Current work status	%
Currently have a job but actively look for a better one	6
Currently have a job	35
No job currently	56
To be graduated	2

Bureau of Statistics of China. The next table 10 presents the joint distribution of migrants' current residential place (calculated on the province level) and education level. A comparison is made on the joint distribution between the Zhaopin.com resume data sample and the 2015 census data issued by the National Bureau of Statistics of China.

From all above tables, it can be summarized that the online job seekers in our sample represents a sub-population who have higher education level and are gathering more intensively in the eastern coast area of China than the average of the whole population. This bias is reasonable as threshold exists for seeking job online, the online job-seekers have to get used to use computer and internet in their daily life, meanwhile they own stable access to the internet. Therefore, online job seekers can only be those relatively high-educated workers and stays in relatively developed region of China (most of the workers in remote regions, such as the Tibet and Qinghai Province, have very limited access to the internet, they are naturally under-represented in our sample). Although bias exists for our sample, it at least represents an important sub-population, the online job-seekers/migrants. As the economic growth and the further development of internet, this sub-population constitutes an increasingly influential portion of the labor supply in the entire Chinese labor market, therefore, concentrating on this sub-population is meaningful for interpret the future labor force migration pattern in China.

Table 9: Joint distribution of migrant education and working industry

Industry	Resume sample from Zhaopin.com			Census data 2015		
	% migrants	% migrants with education level no more than high school	% migrants with education level at least profession school	% migrants	% migrants with education level no more than high school	% migrants with education level at least profession school
Transportation, warehousing and postal services	2.7	18	82	3.7	85.3	14.7
Accommodation and Catering	2	17	83	3.9	92.4	7.6
Information Transmission, Software and Information Technology Services	12.5	7.7	92.3	0.9	30.9	69.1
Public Management, Social Security and Social Organization	2.2	8.6	91.4	3.3	42.4	57.6
Agriculture, forestry, animal husbandry and fishery	0.8	10.5	89.5	37	99.4	0.6
Manufacturing	18.3	11.6	88.4	18.1	88.3	11.7
Health and Social Work	1.1	13.7	86.3	1.6	39.8	60.2
Residential services, repairs and other services	3.8	11.6	88.4	2.6	92.4	7.6
Construction Industry	4.6	9	91	7.4	92.4	7.6
Real Estate	2.3	13.8	86.2	1	69.5	30.5
Wholesale and Retail	15.4	9.9	90.1	12.2	84.5	15.5
Education	3.4	7.9	92.1	2.8	30.9	69.1
Entertainment, Sport and Cultural Industry	4.8	10.2	89.8	0.5	63	37
Water Conservancy, Environment and Public Facilities Management	1.3	8.8	91.2	0.4	77.8	22.2
Electricity, Heat, Gas and water production and supply	1.5	9.3	90.7	0.8	60.4	39.6
Scientific Research and Technological Services	1.6	9.6	90.4	0.5	35.2	64.8
Rental and Business Services	7.5	10.2	89.8	1.2	59.3	40.7
Mining Industry	3.1	7.8	92.2	1.1	81.1	18.9
Financial Industry	5.8	8.2	91.8	1.2	31.7	68.3
Others	5.3	13.3	86.7	-	-	-

Table 10: Joint distribution of migrant education and current residential place

Province	Resume sample from Zhaopin.com			Census data 2015		
	% migrants	% migrants with education level no more than high school	% migrants with education level at least profession school	% migrants	% migrants with education level no more than high school	% migrants with education level at least profession school
Shanghai	12.4	12	88	1.8	63.1	36.9
Yunnan	0.2	18.9	81.1	3.8	89.7	10.3
Neimenggu	0.3	6.9	93.1	1.8	82.8	17.2
Beijing	23	10.3	89.7	1.5	51.7	48.3
Jilin	3.9	12.9	87.1	2	87.1	12.9
Sichuan	6.4	10.5	89.5	6.1	89.1	10.9
Tianjin	2.6	8.7	91.3	1.1	74.3	25.7
Ningxia	0.1	20.7	79.3	0.5	84.5	15.5
Anhui	1.1	10.2	89.8	4.5	89.8	10.2
Shandong	4.3	9.1	90.9	7.8	87.7	12.3
Shanxi	0.3	9.2	90.8	2.4	84.4	15.6
Guangdong	10.8	13.1	86.9	8.5	86.7	13.3
Guangdong	1.1	11.9	88.1	3.4	90.5	9.5
Xinjiang	0.2	08.3	91.7	1.7	82.2	17.8
Jiangsu	5.7	11.5	88.5	6.4	83	17
Jiangxi	1.2	11.2	88.8	3.2	89.8	10.2
Hebei	1.5	8.7	91.3	5.4	89.1	10.9
Henan	1.4	10	90	6.3	91.5	8.5
Zhejiang	9.1	9.8	90.2	4.4	84.8	15.2
Hainan	0.2	15.9	84.1	0.6	86.8	13.2
Hubei	1.5	9	91	4.1	86.7	13.3
Hunan	1.4	14.1	85.9	4.8	87.4	12.6
Gansu	0.1	8.8	91.2	1.9	85	15
Fujian	0.6	19.7	80.3	2.8	84.1	15.9
Tibet	¡0.1	0	1	0.3	93.9	6.1
Guizhou	0.2	19.7	80.3	2.2	89.9	10.1
Liaoning	4.5	8.7	91.3	3.1	81	19
Chongqing	1.6	12.6	87.4	2.1	86.8	13.2
Shanxi	0.7	12.3	87.7	2.5	86	14
Qinghai	<0.1	14.3	85.7	0.4	88	12
Heilongjiang	3.5	8.5	91.5	2.6	87.8	12.2
Hongkong, Maco, Taiwan and others	1.9	13.9	86.1	-	-	-

Type-specific migration networks and decentralization trend

The difference migration network formed by entry-wisely subtracting the type-specific migration probability from the overall migration probability is presented in Fig. 2 for three migrant groups that have undergruate degree, monthly salary 84,000 and are younger than 20-year-old respectively. The three types are selected because they deviate from the overall migration trend most significantly at stage i). In 2a and 2b, the difference migration network by the undergraduate group of migrants is plotted, it shows that the undergraduate migrants originated from "other cities" are more likely than the average to migrate into the central cities such as Beijing in the stage i) and Shanghai in the stage ii) both of which are the top cities in China and pointed toward by the coarse blue arrows in Fig. 2a and 2b respectively. This observation implies that the high-educated migrants have extra preference to top cities which agrees with the findings in ref [1, 2]. For the high-income migrants with monthly salary 84,000, it is shown in Fig. 2d that the strong migration tendency from the top city, Shanghai, and a couple of local central cities, such as Shenyang, Yinchuan and Lanzhou, to the "other cities" exists in the stage ii) migration. This observation is very special, it does neither exist for the other migrant types in Fig. 2 nor is documented in the existing studies as far as we know. One possible explanation is the return migration trend [3, 4] after the financial crisis in 2008, which means the high-income migrants intend to move back to somewhere close to their home town so as to chase better life quality rather than keep accumulating material wealth in rich regions. Finally, as shown in Fig. 2e and 2f, the migrants younger than 20-year-old are more likely than the average to migrate from the other cities to the city clusters centered at Guangzhou, Fuzhou, Changsha and so on, rather than the top cities, e.g. Beijing and Shanghai. This observation holds for both migration stages which might be caused by the fact that younger migrants are less capable of finding well-paid job opportunities, thus are less likely to survive in the top cities as the top cities are also the most expansive cities[2, 5].

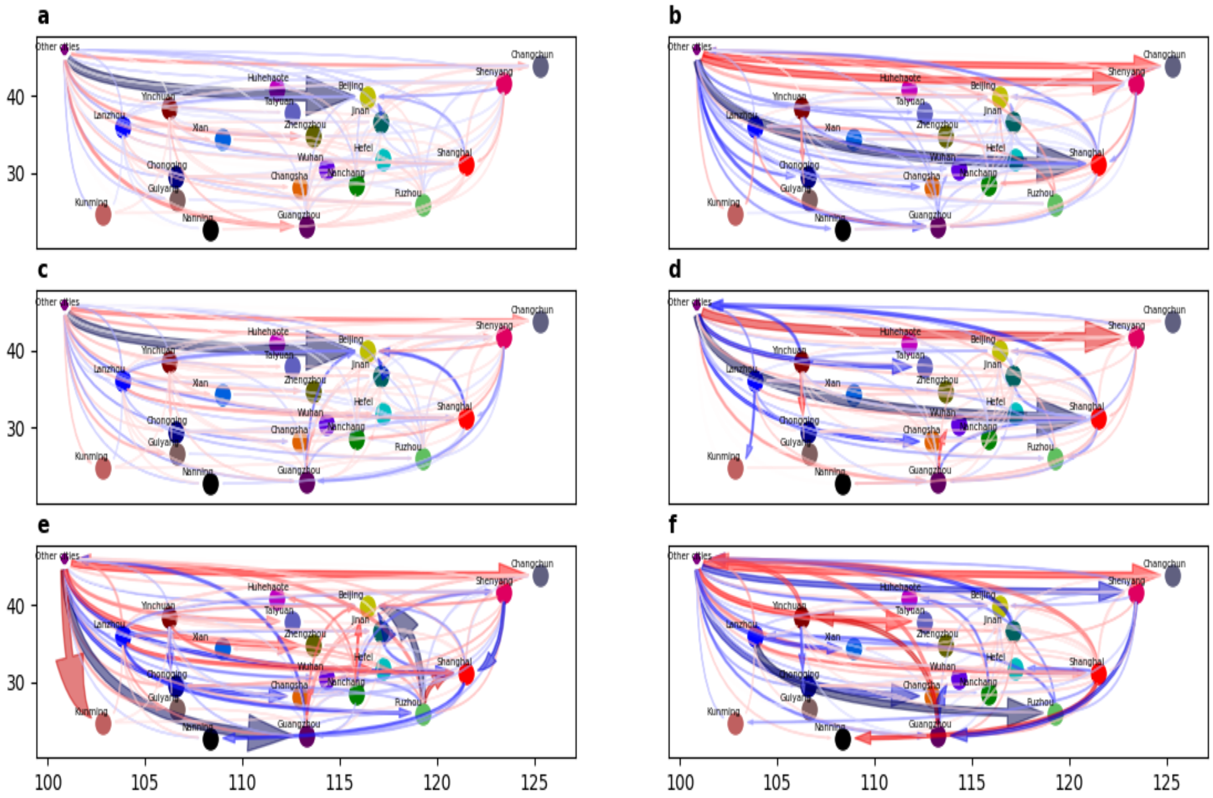

Figure 1: Two-stage difference migration networks for three migrant-types with the most significant deviation at stage stage i)

For different education, income and age types, the difference migration networks are plotted for the types that deviate most significantly from the whole population at the migration stage i) in terms of their type-specific migraiton network. Based on the discussion in the main manuscript, the three types are the undergraduate type for education, the type with monthly salary 84,000 for income and the 10-20-year-old type for age, respectively, which are all significant at least the 0.05 confidential level. The difference network is computed by subtracting the type-specific network from the the overall migration network. In **a**, **c** and **e**, the difference network for stage i) migration are plotted for undergraduate, 84,000-monthly-salary and 10-20-year-old migrants, respecitvely. In **b**, **d** and **f** the stage ii) difference migration networks are plotted for the same three migrant types. In all the figures, the arrows are always pointed toward the destination, the size, opacity and darkness of arrows represent the absolute value of the link weight in the difference network, the red-colored arrow represents on which the overall migration probabilty is greater than the type-specific migration probability while the blue-colored arrow represents the opposite on which the type-specific migration probability is greater than the overall probability.

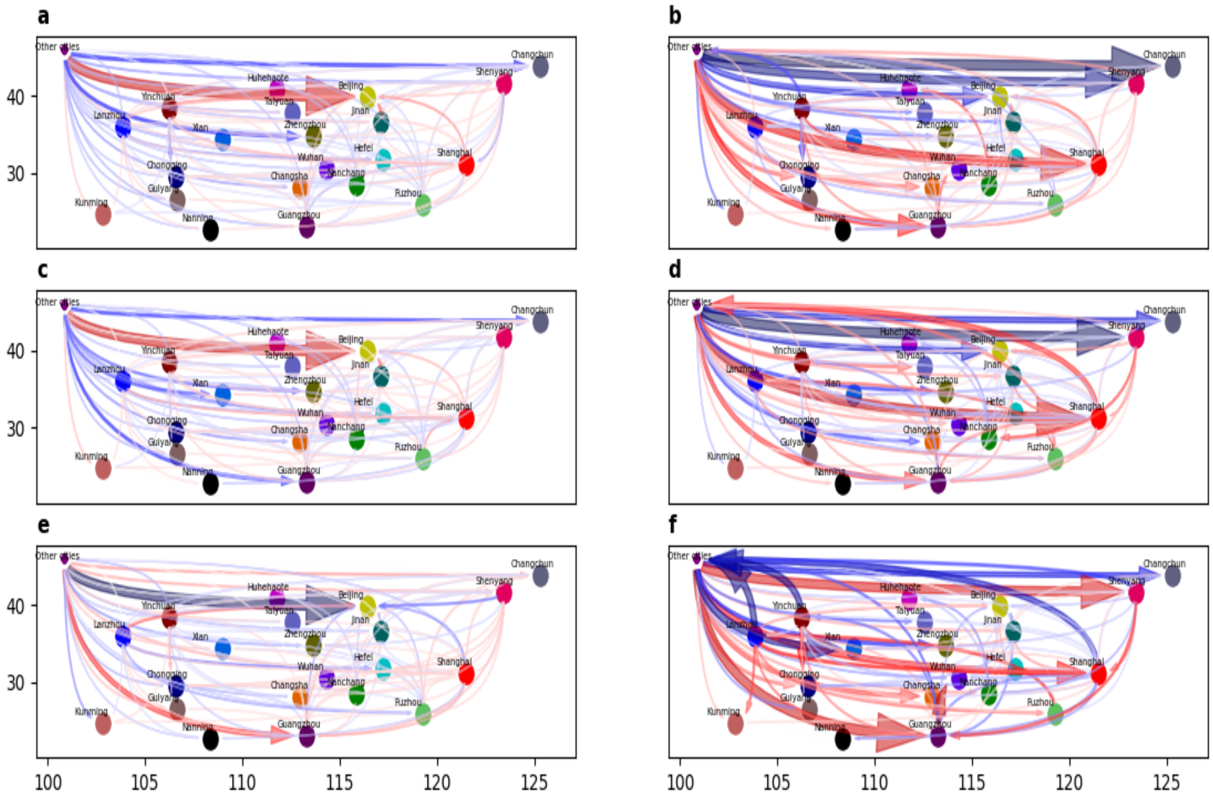

Figure 2: Two-stage difference migration networks for three migrant types with the most significant deviation at stage ii)

For different education, income and age types, the difference migration networks are plotted for the types that deviate most significantly from the whole population at the migration stage ii) in terms of their type-specific migraiton network. Based on the discussion in the main manuscript, the three types are the type of professional school degree for education, the type with monthly salary 6,000 for income and the 30-40-year-old type for age, respectively, which are all significant at least the 0.05 confidential level. The difference network is computed by subtracting the type-specific network from the the overall migration network. In **a**, **c** and **e**, the difference network for stage i) migration are plotted for undergraduate, 84,000-monthly-salary and 10-20-year-old migrants, respecitvely. In **b**, **d** and **f** the stage ii) difference migration networks are plotted for the same three migrant types. In all the figures, the arrows are always pointed toward the destination, the size, opacity and darkness of arrows represent the absolute value of the link weight in the difference network, the red-colored arrow represents on which the overall migration probabilty is greater than the type-specific migration probability while the blue-colored arrow represents the opposite on which the type-specific migration probability is greater than the overall probability.

Hypothesis test for city features

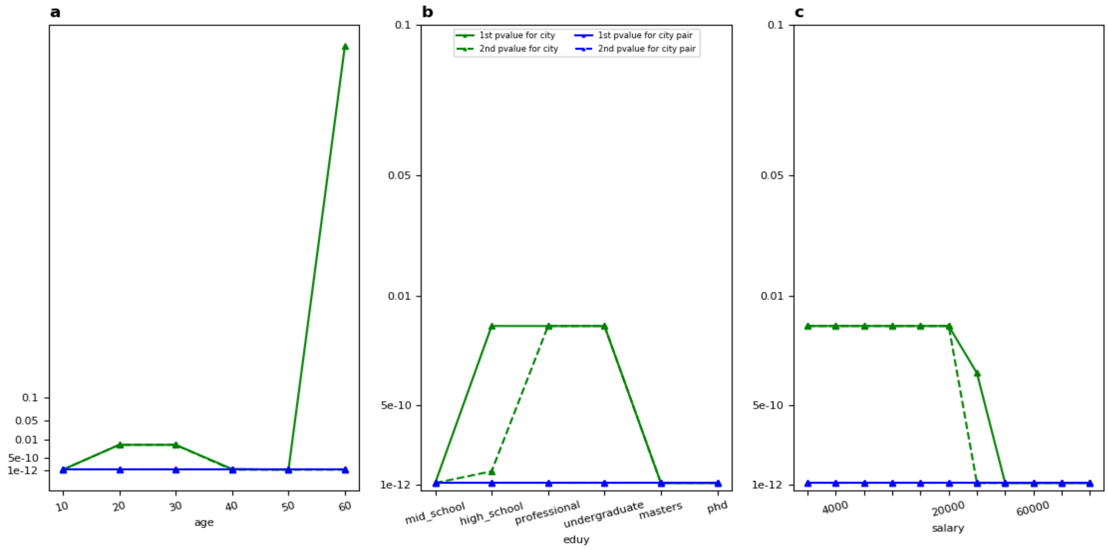

Figure 3: Test of the difference of education resources between **np** and **nn** classes

a, **b** and **c** present the left-tail pvalues of the null hypothesis that there is no difference in the feature “teacher’s number” between the **np** and **nn** class of cities (city-pairs) for a variety of age, education and income types. The smaller pvalue implies the greater confidence to reject the null hypothesis and take the alternative hypothesis that the **np** class has much higher teacher’s number than the **nn** class.

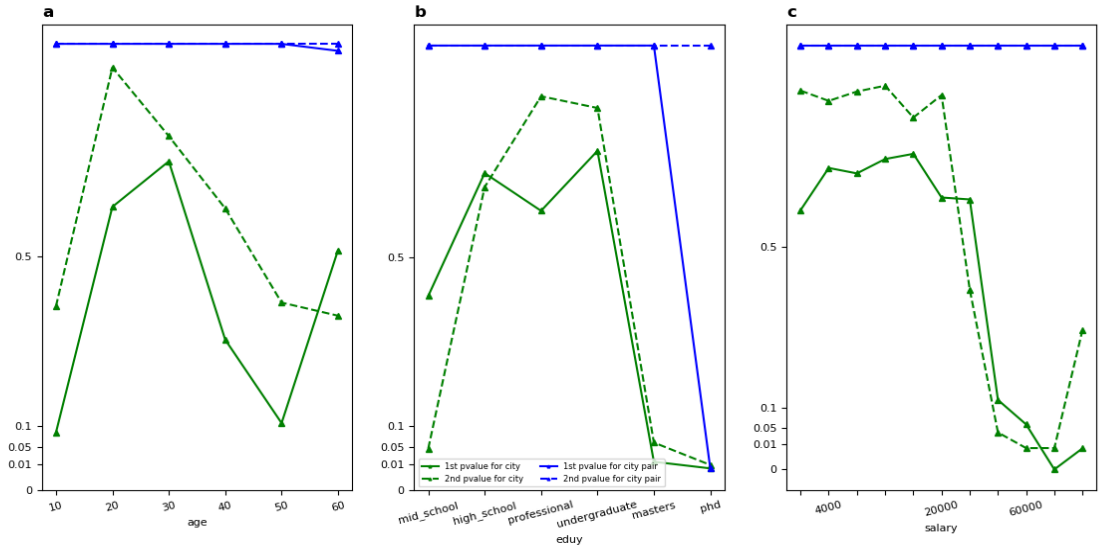

Figure 4: Test of the difference of population between **np** and **nn** classes

a, **b** and **c** present the left-tail pvalues of the null hypothesis that there is no difference in the feature "population" between the **np** and **nn** class of cities (city-pairs) for a variety of age, education and income types. The smaller pvalue implies the greater confidence to reject the null hypothesis and take the alternative hypothesis that the **np** class has much greater population size than the **nn** class.

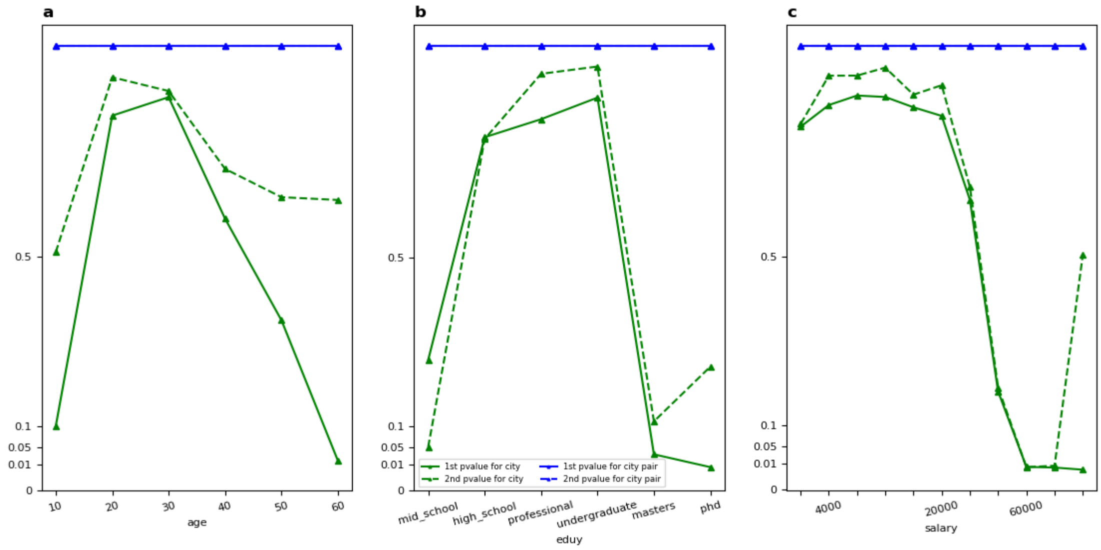

Figure 5: Test of the difference of GDP between **np** and **nn** classes

a, **b** and **c** present the left-tail pvalues of the null hypothesis that there is no difference in the feature "GDP" between the **np** and **nn** class of cities (city-pairs) for a variety of age, education and income types. The smaller pvalue implies the greater confidence to reject the null hypothesis and take the alternative hypothesis that the **np** class has much greater GDP than the **nn** class.

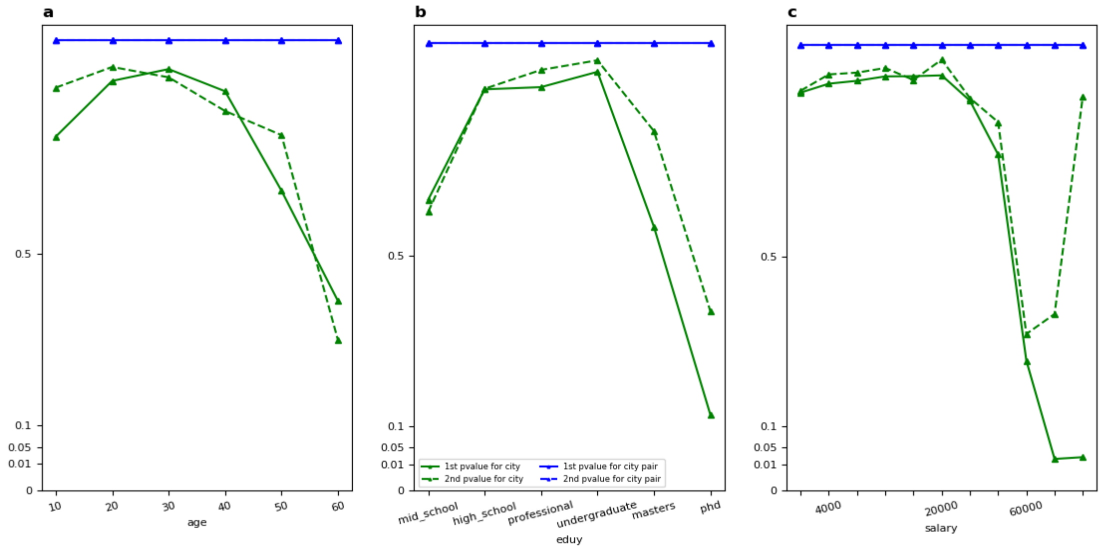

Figure 6: Test of the difference of FDI between **np** and **nn** classes

a, **b** and **c** present the left-tail pvalues of the null hypothesis that there is no difference in the feature "FDI" between the **np** and **nn** class of cities (city-pairs) for a variety of age, education and income types. The smaller pvalue implies the greater confidence to reject the null hypothesis and take the alternative hypothesis that the **np** class has much greater FDI than the **nn** class.

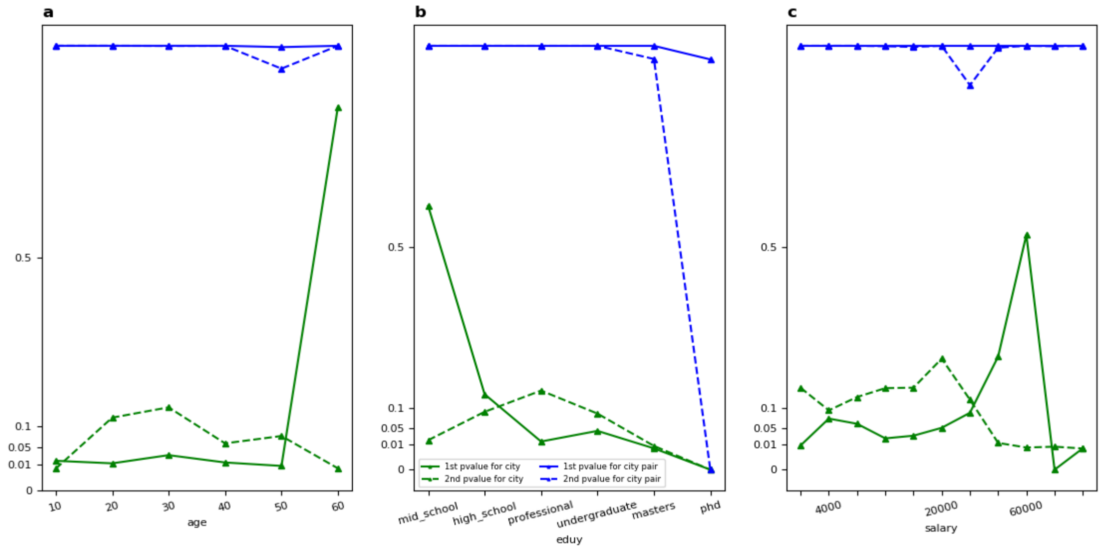

Figure 7: Test of the difference of road length between **np** and **nn** classes

a, **b** and **c** present the left-tail pvalues of the null hypothesis that there is no difference in the feature "teacher's number" between the **np** and **nn** class of cities (city-pairs) for a variety of age, education and income types. The smaller pvalue implies the greater confidence to reject the null hypothesis and take the alternative hypothesis that the **np** class has much longer road length than the **nn** class.

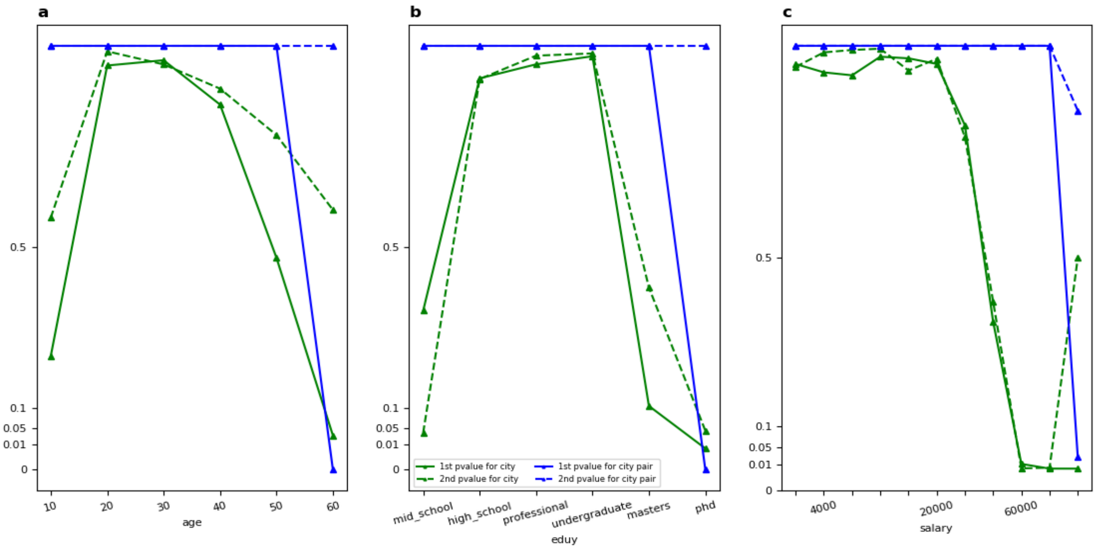

Figure 8: Test of the difference of built-up area between **np** and **nn** classes

a, **b** and **c** present the left-tail pvalues of the null hypothesis that there is no difference in the feature "bulit-up area" between the **np** and **nn** class of cities (city-pairs) for a variety of age, education and income types. The smaller pvalue implies the greater confidence to reject the null hypothesis and take the alternative hypothesis that the **np** class has much larger built-up area than the **nn** class.

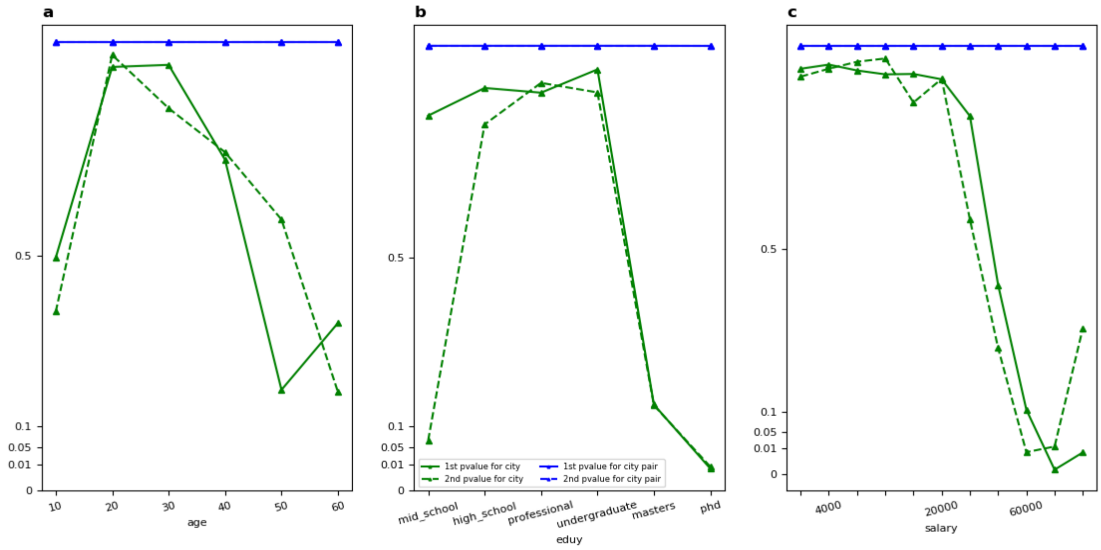

Figure 9: Test of the difference of hospital bed number between **np** and **nn** classes

a, **b** and **c** present the left-tail pvalues of the null hypothesis that there is no difference in the feature "hospital bed number" between the **np** and **nn** class of cities (city-pairs) for a variety of age, education and income types. The smaller pvalue implies the greater confidence to reject the null hypothesis and take the alternative hypothesis that the **np** class has much more hospital bed number than the **nn** class.

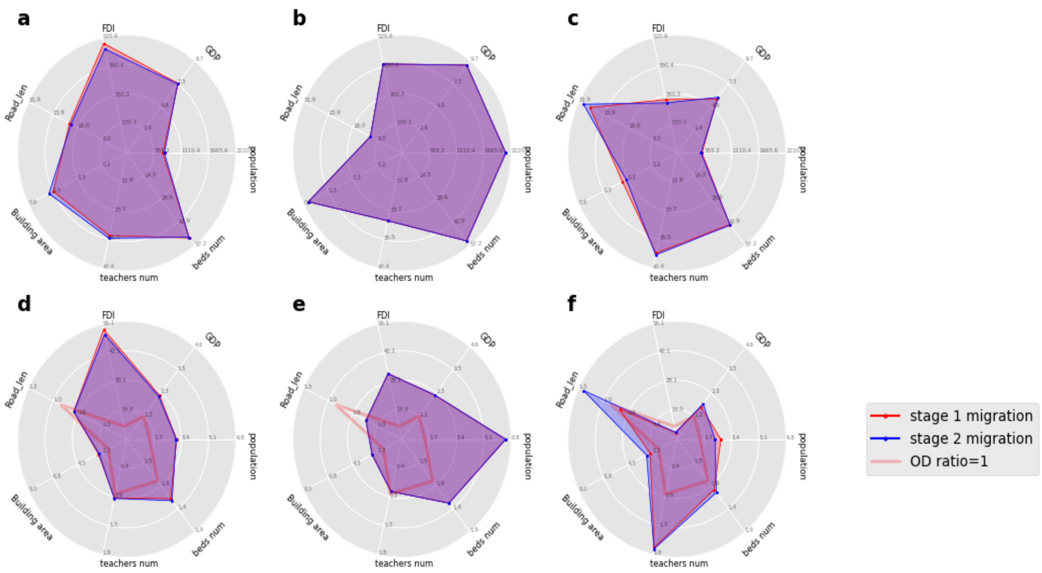

Figure 10: Mean per capita features of city classes

a, **b** and **c** present the radial plot for the means of the population size and the per capita value of the other six city-level features for the **nn**, **pn** and **np** class of cities respectively for both stages of migration; **d-f** present the radial plot for the mean OD ratios of the seven city-level features for the **nn**, **pn** and **np** class of city pairs respectively for the two stages of migration. Because the **pp** class contains no city nor city pair for both stage i) and ii) migration, the relevant radial plots are missing. During the classification, to avoid the data noise, we trimed those very small valued $DE^1_{x,ij}$ and $DE^2_{x,ij}$ in the sense of setting $DE^1_{x,ij}(DE^2_{x,ij})$ as 0 when their absolute value is less than 0.001, then the resulting city pair ij is discarded as noisy point and won't be rendered into any of the four classes.

References

[1] Liu Y., Stillwell J., Shen J. and Daras K. Interprovincial migration, regional development and state policy in China, 1985–2010. *Applied Spatial Analysis and Policy*, 7(1): 47–70, 2014.

[2] Liu Y., Shen J., Xu W. and Wang G. From school to university to work: migration of highly educated youths in China. *The Annals of Regional Science*, 59(3): 651–676, 2017.

[3] Zhao L. Return Migration in ChinaTrends and Impacts. *Changing State-Society Relations in CONTEMPORARY CHINA*, 3–18, 2017.

[4] Chen J. and Cui C. Large City or Small Town: an Empirical Analysis of the Effects

of Migration Strategies of Rural Households on Income in China. *Applied Research in Quality of Life*, 13(1): 211–228, 2018.

[5] Shen J. and Liu Y. Skilled and less-skilled interregional migration in China: A comparative analysis of spatial patterns and the decision to migrate in 2000–2005. *Habitat International*, 57: 1–10, 2016.
